# Robustness and Strategies of Adaptation among Farmer Varieties of African Rice (*Oryza glaberrima*) and Asian Rice (*Oryza sativa*) across West Africa

**DOI:** 10.1371/journal.pone.0034801

**Published:** 2013-03-01

**Authors:** Alfred Mokuwa, Edwin Nuijten, Florent Okry, Béla Teeken, Harro Maat, Paul Richards, Paul C. Struik

**Affiliations:** 1 Technology and Agrarian Development group, Wageningen University, Wageningen, The Netherlands; 2 Africa Rice Center, Cotonou, Bénin; 3 Centre for Crop Systems Analysis, Wageningen University, Wageningen, The Netherlands; New York State Museum, United States of America

## Abstract

This study offers evidence of the robustness of farmer rice varieties (*Oryza glaberrima* and *O. sativa*) in West Africa. Our experiments in five West African countries showed that farmer varieties were tolerant of sub-optimal conditions, but employed a range of strategies to cope with stress. Varieties belonging to the species *Oryza glaberrima* – solely the product of farmer agency – were the most successful in adapting to a range of adverse conditions. Some of the farmer selections from within the *indica* and *japonica* subspecies of *O. sativa* also performed well in a range of conditions, but other farmer selections from within these two subspecies were mainly limited to more specific niches. The results contradict the rather common belief that farmer varieties are only of local value. Farmer varieties should be considered by breeding programmes and used (alongside improved varieties) in dissemination projects for rural food security.

## Introduction

It is often supposed that crops should only be grown where conditions are favourable. This is not an option for farmers cultivating food crops with limited resources. They have to grow what they need with the conditions they have been given. In short, they have to cope with sub-optimality. For these farmers, adaptability of varieties under sub-optimal conditions is an essential requirement [1], [2]. Hypothetically, we should expect to find this adaptability among farmer varieties since these are to a large extent the product of farmer selection. This would mean that farmer varieties are the result of interplay between local ecological and social factors.

In large parts of West Africa small-scale farmers rely upon the cultivation of upland rice under low input conditions in a great diversity of micro-environments. The first rice farming in West Africa was based exclusively on African rice (*O. glaberrima* Steud.). The cultivation of African rice is entirely a result of farmer agency as African rice has never been disseminated by extension programmes. Asian rice (*Oryza sativa*) is a more recent introduction, perhaps during the period of the Atlantic Slave trade (beginning c. 1550), or earlier via trans-Saharan trade routes. Asian rice has two main subspecies: *Oryza sativa* var. *japonica* (short-grained, mainly grown as upland rice) and *O. sativa* var. *indica* (long-grained, mainly a lowland type).

Today, farmers in the region mainly grow the two types of Asian rice. Nevertheless in certain areas African rice remains an important crop type [2]–[6]. These areas all seem to have a shared history of rice cultivation taking place against a background of special difficulty, such as war, population displacement or harsh ecological conditions [7]. This suggests the species may be selected for its greater tolerance to sub-optimal conditions when compared to Asian rice. The logic of the present study, therefore, is to compare African and Asian rice, in farmer conditions, in order to understand the extent to which plasticity and adaptability are factors in farmer varietal choice. The overall aim of the study is to secure a better knowledge base for possible complementary strategies of variety promotion. These complementary strategies would give due consideration both to varieties developed through scientific research and varieties produced by farmer selection. The objective is to assess the case for protecting farmer varieties as an important aspect of local food security, in an environment in which development agencies seek more generally to expand the range of high-yielding cultivars to meet urban rice demand across the region. Our study reports on differences in response to varying environments of a large sample of farmer varieties across five West African countries in the high-rainfall coastal zone.

The study tests the hypothesis that African rice may be more robust than Asian rice in West African farmer conditions. Here robustness is seen as the ability of a variety or group of varieties to perform well in a diversity of cultivation conditions.

The following research questions are posed:

1. Are farmer varieties of *O. glaberrima* better suited to sub-optimal agro-ecological conditions than varieties of the two subspecies of *O. sativa*?
2. Do farmer varieties of *O. glaberrima* adapt better to different environmental conditions than varieties of the two subspecies of *O. sativa*?
3. What are the physiological processes and social and eco-regional patterns underlying the adaptation of farmer varieties across environments?

In achieving robustness, varieties can respond to environmental conditions by showing phenotypic plasticity in a range of traits [8], [9]. Different varieties or groups of varieties achieve robustness by combining variability and stability of different traits, thus constituting different physiological strategies. Hence, this study investigates whether different botanical groups of rice, or certain groups of varieties within those botanical groups, have developed different physiological strategies to achieve adaptation.

The hypothesis that African rice might be more robust than Asian rice in West African conditions would make sense of a number of observations already reported. Richards [7] has offered some general evidence that African rice is an important food reserve for communities facing special difficulty (e.g. when displaced by war). Dingkuhn et al. [10] and Johnson et al. [11] showed evidence that *O. glaberrima* has a vegetative vigour superior to that of *O. sativa*, thus is better able to suppress weeds. Sumi and Katayama [12] provided evidence that African rice has a yield potential similar to Asian counterparts.

For a proper understanding of the paper we offer the following definitions of concepts and notions.

### Robustness

The persistence of a system's characteristic behaviour under sub-optimal conditions, implying stable performance across environments. In the context of this paper, robustness is taken to be the ability of a variety or a group of varieties to yield well across distinct environments.

### Adaptability

The ability of a variety or a group of varieties to be robust. Adaptability implies significant Genotype (G) × Environment (E) interactions.

Plasticity: the physiological process through which varieties adjust their phenotypes in response to different environmental conditions [13]. A plastic response of this nature does not require changes in gene frequencies (i.e. evolution). Such phenotypic shifts can allow varieties to achieve adaptability [9].

### Sub-optimal farming

characterised by no or limited mineral fertilisation, no or natural pest and disease control, rain fed moisture conditions, rarely mono cropping, and below an optimal or standard level of output.

### Tolerance

The ability of a variety to survive adverse conditions with only a small reduction in performance.

## Results

In the following sections ten relevant variables are investigated for each botanical group (*glaberrima*, *indica* or *japonica*) and molecular cluster (see section on Materials and Methods). These ten variables were used to analyse the vegetative growth and yield components (see section on Materials and methods): maximum canopy cover (V_max_; %), accumulated canopy cover (A; %.day), plant height (cm), number of tillers per plant (# tillers), days to 50% flowering (50% flowering), number of panicles per plant (# panicles), panicle length (cm), panicle weight (g), 200 grain weight (g) and grain yield (kg/ha). The variables are dealt with one by one and cross references are made among them to unravel strategies of adaptation. Graphs are used to compare performance of each variable across environments. ANOVAs provided important information on adaptability, as they provided estimates of G×E interactions (Tables 1–10).

**Table 1:** Main effects of and interactions between genotype, sowing date and trial location regarding crop characteristics, including maximum canopy development (V_max_), accumulated canopy (A), plant height, number of tillers per plant (# Tillers), days to 50% flowering (50% Flowering), number of panicles per plant (# Panicles), panicle length, panicle weight, 200 grain weight and yield of 24 genotypes (all botanical groups and molecular clusters together).

Variable	Genotype	Sowing	Location	Genotype × Sowing	Genotype × Location	Sowing × Location	Genotype × Sowing × Location
**V_max_^d^**	0.000***	0.758	0.026*	0.092	0.881	0.029*	-
**A ^d^**	0.000***	0.435	0.027*	0.014*	0.444	0.001***	-
**Plant height ^f^**	0.000***	0.922	0.002**	0.612	0.000***	0.000***	0.264
**# Tillers ^f^**	0.000***	0.533	0.006**	0.043*	0.000***	0.000***	0.986
**50% Flowering ^f^**	0.000***	0.011*	0.000***	0.008**	0.000***	0.003**	0.000***
**# Panicles ^a^**	0.000***	0.334	0.112	0.005**	0.000***	0.000***	0.947
**Panicle length ^a^**	0.000***	0.890	0.003**	0.023*	0.000***	0.000***	0.017*
**Panicle weight ^e^**	0.000***	0.140	0.502	0.236	0.157	0.194	0.012*
**200 grain weight ^b^**	0.000***	0.318	0.006**	0.069	0.018*	0.031*	0.850
**Yield ^c^**	0.000***	0.070	0.042*	0.583	0.873	0.020*	0.000***

Values in the table are p values (three-way ANOVA). *: Significant at 0.05 level. **: Significant at 0.01 level. ***: Significant at 0.001 level. a: ANOVA performed for Guinea Bissau, Guinea and Sierra Leone. b: ANOVA performed for Guinea Bissau, Guinea, Ghana and Togo. c: ANOVA performed for Guinea Bissau, Ghana, Sierra Leone and Togo. d: ANOVA performed for Ghana, Guinea and Togo. e: ANOVA performed for Ghana and Togo. f: ANOVA performed for all five countries. -: not assessed.

**Table 2:** Main effects of and interactions between genotype, sowing date and trial location regarding crop characteristics, including maximum canopy development (V_max_), accumulated canopy (A), plant height, number of tillers per plant (# Tillers), days to 50% flowering (50% Flowering), number of panicles per plant (# Panicles), panicle length, panicle weight, 200 grain weight and yield of the *Glaberrima* botanical group.

Variable	Genotype	Sowing	Location	Genotype × Sowing	Genotype × Location	Sowing × Location	Genotype × Sowing × Location
**V_max_^d^**	0.190	0.373	0.083	0.464	0.319	0.000***	-
**A ^d^**	0.260	0.217	0.055	0.268	0.132	0.000***	-
**Plant height ^f^**	0.000***	0.797	0.009**	0.471	0.001***	0.000***	0.469
**# Tillers ^f^**	0.097	0.246	0.003**	0.268	0.000***	0.014*	0.612
**50% Flowering ^f^**	0.000***	0.007**	0.001***	0.069	0.014*	0.024*	0.000***
**# Panicles ^a^**	0.314	0.267	0.117	0.025*	0.000***	0.000***	0.998
**Panicle length ^a^**	0.000***	0.810	0.001***	0.024*	0.004**	0.009**	0.024*
**Panicle weight ^e^**	0.051	0.255	0.081	0.359	0.088	0.279	0.563
**200 grain weight ^b^**	0.000***	0.457	0.003**	0.584	0.019*	0.103	0.940
**Yield ^c^**	0.000***	0.458	0.254	0.619	0.981	0.002**	0.000***

Values in the table are p values (three-way ANOVA). *: Significant at 0.05 level. **: Significant at 0.01 level. ***: Significant at 0.001 level. a: ANOVA performed for Guinea Bissau, Guinea and Sierra Leone. b: ANOVA performed for Guinea Bissau, Guinea, Ghana and Togo. c: ANOVA performed for Guinea Bissau, Ghana, Sierra Leone and Togo. d: ANOVA performed for Ghana, Guinea and Togo. e: ANOVA performed for Ghana and Togo. f: ANOVA performed for all five countries. -: not assessed.

**Table 3:** Main effects of and interactions between genotype, sowing date and trial location regarding crop characteristics, including maximum canopy development (V_max_), accumulated canopy (A), plant height, number of tillers per plant (# Tillers), days to 50% flowering (50% Flowering), number of panicles per plant (# Panicles), panicle length, panicle weight, 200 grain weight and yield of the cluster of *Glaberrima* from Lower Guinea Coast (Glab_LowerCoast).

Variable	Genotype	Sowing	Location	Genotype × Sowing	Genotype × Location	Sowing × Location	Genotype × Sowing × Location
**V_max_^d^**	0.137	0.737	0.176	0.330	0.877	0.172	-
**A ^d^**	0.740	0.464	0.082	0.129	0.609	0.053	-
**Plant height ^f^**	0.567	0.566	0.218	0.685	0.665	0.641	0.042*
**# Tillers ^f^**	0.852	0.061	0.002**	0.638	0.026*	0.347	0.935
**50% Flowering ^f^**	0.014*	0.001***	0.004**	0.086	0.061	0.534	0.022*
**# Panicles ^a^**	0.840	0.243	0.086	0.145	0.091	0.008**	0.963
**Panicle length ^a^**	0.582	0.164	0.178	0.144	0.791	0.441	0.393
**Panicle weight ^e^**	0.274	0.081	0.370	0.641	0.330	0.926	0.517
**200 grain weight ^b^**	0.056	0.421	0.119	0.654	0.325	0.258	0.218
**Yield ^c^**	0.099	0.316	-	0.570	0.899	0.604	0.017*

Values in the table are p values (three-way ANOVA). *: Significant at 0.05 level. **: Significant at 0.01 level. ***: Significant at 0.001 level. a: ANOVA performed for Guinea Bissau, Guinea and Sierra Leone. b: ANOVA performed for Guinea Bissau, Guinea, Ghana and Togo. c: ANOVA performed for Guinea Bissau, Ghana, Sierra Leone and Togo. d: ANOVA performed for Ghana, Guinea and Togo. e: ANOVA performed for Ghana and Togo. f: ANOVA performed for all five countries. -: not assessed.

**Table 4:** Main effects of and interactions between genotype, sowing date and trial location regarding crop characteristics, including maximum canopy development (V_max_), accumulated canopy (A), plant height, number of tillers per plant (# Tillers), days to 50% flowering (50% Flowering), number of panicles per plant (# Panicles), panicle length, panicle weight, 200 grain weight and yield of the cluster of *Glaberrima* from Upper Guinea Coast (Glab_UpperCoast).

Variable	Genotype	Sowing	Location	Genotype × Sowing	Genotype × Location	Sowing × Location	Genotype × Sowing × Location
**V_max_^d^**	0.589	0.276	0.076	0.973	0.178	0.001***	-
**A ^d^**	0.545	0.170	0.055	0.667	0.184	0.002**	-
**Plant height ^f^**	0.003**	0.702	0.027*	0.209	0.000***	0.000***	0.956
**# Tillers ^f^**	0.664	0.397	0.031*	0.270	0.008**	0.056	0.145
**50% Flowering ^f^**	0.000***	0.017*	0.005**	0.455	0.290	0.091	0.000***
**# Panicles ^a^**	0.372	0.294	0.144	0.025*	0.000***	0.000***	0.982
**Panicle length ^a^**	0.018*	0.919	0.010**	0.003**	0.000***	0.000***	0.439
**Panicle weight ^e^**	0.309	0.300	0.242	0.322	0.128	0.221	0.454
**200 grain weight ^b^**	0.202	0.581	0.001***	0.464	0.013*	0.329	0.980
**Yield ^c^**	0.000***	0.519	0.412	0.344	0.902	0.001***	0.039*

Values in the table are p values (three-way ANOVA). *: Significant at 0.05 level. **: Significant at 0.01 level. ***: Significant at 0.001 level. a: ANOVA performed for Guinea Bissau, Guinea and Sierra Leone. b: ANOVA performed for Guinea Bissau, Guinea, Ghana and Togo. c: ANOVA performed for Guinea Bissau, Ghana, Sierra Leone and Togo. d: ANOVA performed for Ghana, Guinea and Togo. e: ANOVA performed for Ghana and Togo. f: ANOVA performed for all five countries. -: not assessed.

**Table 5:** Main effects of and interactions between genotype, sowing date and trial location regarding crop characteristics, including maximum canopy development (V_max_), accumulated canopy (A), plant height, number of tillers per plant (# Tillers), days to 50% flowering (50% Flowering), number of panicles per plant (# Panicles), panicle length, panicle weight, 200 grain weight and yield of the *Indica* botanical group.

Variable	Genotype	Sowing	Location	Genotype × Sowing	Genotype × Location	Sowing × Location	Genotype × Sowing × Location
**V_max_^d^**	0.017*	0.931	0.060	0.160	0.746	0.171	-
**A ^d^**	0.031*	0.588	0.038*	0.177	0.508	0.055	-
**Plant height ^f^**	0.089	0.591	0.000***	0.720	0.000***	0.010**	0.057
**# Tillers ^f^**	0.553	0.998	0.001***	0.022*	0.001***	0.006**	0.979
**50% Flowering ^f^**	0.027*	0.005**	0.000***	0.233	0.003**	0.432	0.120
**# Panicles ^a^**	0.358	0.654	0.149	0.100	0.002**	0.315	0.829
**Panicle length ^a^**	0.162	0.474	0.002**	0.595	0.063	0.377	0.047*
**Panicle weight ^e^**	0.174	0.029*	0.230	0.377	0.271	0.732	0.457
**200 grain weight ^b^**	0.001***	0.053	-	0.339	0.794	0.866	0.365
**Yield ^c^**	0.001***	0.002**	0.358	0.630	0.441	0.916	0.000***

Values in the table are p values (three-way ANOVA). *: Significant at 0.05 level. **: Significant at 0.01 level. ***: Significant at 0.001 level. a: ANOVA performed for Guinea Bissau, Guinea and Sierra Leone. b: ANOVA performed for Guinea Bissau, Guinea, Ghana and Togo. c: ANOVA performed for Guinea Bissau, Ghana, Sierra Leone and Togo. d: ANOVA performed for Ghana, Guinea and Togo. e: ANOVA performed for Ghana and Togo. f: ANOVA performed for all five countries. -: not assessed.

**Table 6:** Main effects of and interactions between genotype, sowing date and trial location regarding crop characteristics, including maximum canopy development (V_max_), accumulated canopy (A), plant height, number of tillers per plant (# Tillers), days to 50% flowering (50% Flowering), number of panicles per plant (# Panicles), panicle length, panicle weight, 200 grain weight and yield of the cluster of *Indica* from Ghana (Ind_Gh).

Variable	Genotype	Sowing	Location	Genotype × Sowing	Genotype × Location	Sowing × Location	Genotype × Sowing × Location
**V_max_^d^**	0.057	0.362	estimate	0.229	0.943	0.756	-
**A ^d^**	0.099	0.762	0.439	0.253	0.891	0.370	-
**Plant height ^f^**	0.385	0.480	0.001 ***	0.798	0.022*	0.124	0.012*
**# Tillers ^f^**	0.361	0.580	0.005 **	0.078	0.055	0.201	0.702
**50% Flowering ^f^**	0.026*	0.026*	0.011*	0.245	0.172	0.539	0.019*
**# Panicles ^a^**	0.448	0.548	0.864	0.222	0.038*	0.644	0.440
**Panicle length ^a^**	0.158	0.872	0.081	0.475	0.170	0.287	0.139
**Panicle weight ^e^**	-	0.119	-	-	-	-	-
**200 grain weight ^b^**	-	-	-	-	-	-	-
**Yield ^c^**	0.016*	0.062	0.061	0.385	0.192	0.342	0.000 ***

Values in the table are p values (three-way ANOVA). *: Significant at 0.05 level. **: Significant at 0.01 level. ***: Significant at 0.001 level. a: ANOVA performed for Guinea Bissau, Guinea and Sierra Leone. b: ANOVA performed for Guinea Bissau, Guinea, Ghana and Togo. c: ANOVA performed for Guinea Bissau, Ghana, Sierra Leone and Togo. d: ANOVA performed for Ghana, Guinea and Togo. e: ANOVA performed for Ghana and Togo. f: ANOVA performed for all five countries. -: not assessed.

**Table 7:** Main effects of and interactions between genotype, sowing date and trial location regarding crop characteristics, including maximum canopy development (V_max_), accumulated canopy (A), plant height, number of tillers per plant (# Tillers), days to 50% flowering (50% Flowering), number of panicles per plant (# Panicles), panicle length, panicle weight, 200 grain weight and yield from the cluster of *Indica* from Guinea (Ind_Gc).

Variable	Genotype	Sowing	Location	Genotype × Sowing	Genotype × Location	Sowing × Location	Genotype × Sowing × Location
**V_max_^d^**	0.103	0.657	0.025*	0.242	0.074	0.033*	-
**A ^d^**	0.052	0.439	0.017*	0.122	0.100	0.035*	-
**Plant height ^f^**	0.962	0.957	0.000***	0.829	0.025*	0.008**	0.964
**# Tillers ^f^**	0.634	0.440	0.018*	0.384	0.006**	0.031*	0.973
**50% Flowering ^f^**	0.286	0.003**	0.029*	0.551	0.118	0.823	0.391
**# Panicles ^a^**	0.500	0.189	0.114	0.774	0.038*	0.242	0.876
**Panicle length ^a^**	0.781	0.369	0.021*	0.416	0.180	0.397	0.368
**Panicle weight ^e^**	0.412	0.032*	0.377	0.336	0.358	0.761	0.540
**200 grain weight ^b^**	0.272	0.481	0.350	0.535	0.573	0.494	0.302
**Yield ^c^**	0.598	0.097	0.090	0.112	0.454	0.022*	0.501

Values in the table are p values (three-way ANOVA). *: Significant at 0.05 level. **: Significant at 0.01 level. ***: Significant at 0.001 level. a: ANOVA performed for Guinea Bissau, Guinea and Sierra Leone. b: ANOVA performed for Guinea Bissau, Guinea, Ghana and Togo. c: ANOVA performed for Guinea Bissau, Ghana, Sierra Leone and Togo. d: ANOVA performed for Ghana, Guinea and Togo. e: ANOVA performed for Ghana and Togo. f: ANOVA performed for all five countries. -: not assessed.

**Table 8:** Main effects of and interactions between genotype, sowing date and trial location regarding crop characteristics, including maximum canopy development (V_max_), accumulated canopy (A), plant height, number of tillers per plant (# Tillers), days to 50% flowering (50% Flowering), number of panicles per plant (# Panicles), panicle length, panicle weight, 200 grain weight and yield of the *Japonica* botanical group.

Variable	Genotype	Sowing	Location	Genotype × Sowing	Genotype × Location	Sowing × Location	Genotype × Sowing × Location
**V_max_^d^**	0.047**	0.178	0.047**	0.703	0.468	0.011**	-
**A ^d^**	0.176	0.318	0.065	0.818	0.285	0.002***	-
**Plant height ^f^**	0.021*	0.562	0.000***	0.846	0.000***	0.001***	0.404
**# Tillers ^f^**	0.000***	0.755	0.033*	0.965	0.008**	0.000***	0.963
**50% Flowering ^f^**	0.001***	0.431	0.005**	0.108	0.007**	0.000***	0.012*
**# Panicles ^a^**	0.010**	0.803	0.653	0.946	0.282	0.020*	0.121
**Panicle length ^a^**	0.000***	0.860	0.038*	0.043*	0.000***	0.000***	0.784
**Panicle weight ^e^**	0.182	0.158	0.405	0.813	0.608	0.368	0.022*
**200 grain weight ^b^**	0.000***	0.197	0.085	0.178	0.936	0.216	0.660
**Yield ^c^**	0.001***	0.006**	estimate	0.644	0.987	0.884	0.000***

Values in the table are p values (three-way ANOVA). *: Significant at 0.05 level. **: Significant at 0.01 level. ***: Significant at 0.001 level. a: ANOVA performed for Guinea Bissau, Guinea and Sierra Leone. b: ANOVA performed for Guinea Bissau, Guinea, Ghana and Togo. c: ANOVA performed for Guinea Bissau, Ghana, Sierra Leone and Togo. d: ANOVA performed for Ghana, Guinea and Togo. e: ANOVA performed for Ghana and Togo. f: ANOVA performed for all five countries. -: not assessed.

**Table 9:** Main effects of and interactions between genotype, sowing date and trial location regarding crop characteristics, including maximum canopy development (V_max_), accumulated canopy (A), plant height, number of tillers per plant (# Tillers), days to 50% flowering (50% Flowering), number of panicles per plant (# Panicles), panicle length, panicle weight, 200 grain weight and yield of the cluster of *Japonica* from Guinea Bissau and Ghana (Jap_GbGh).

Variable	Genotype	Sowing	Location	Genotype × Sowing	Genotype × Location	Sowing × Location	Genotype × Sowing × Location
**V_max_^d^**	0.331	0.116	0.030*	0.637	0.472	0.142	-
**A ^d^**	0.355	0.205	0.028*	0.725	0.347	0.069	-
**Plant height ^f^**	0.080	0.607	0.000***	0.693	0.004**	0.045*	0.229
**# Tillers ^f^**	0.000 ***	0.764	0.035*	0.891	0.714	0.005**	0.661
**50% Flowering ^f^**	0.857	0.574	0.007**	0.851	0.006**	0.000***	0.408
**# Panicles ^a^**	0.027*	0.805	0.466	0.860	0.995	0.106	0.036*
**Panicle length ^a^**	0.005 **	0.808	0.028*	0.014*	0.001***	0.000***	0.835
**Panicle weight ^e^**	0.074	0.188	0.576	0.495	0.547	0.352	0.091
**200 grain weight ^b^**	0.000 ***	0.571	0.129	0.339	0.917	0.278	0.705
**Yield ^c^**	0.856	0.329	0.089	0.442	0.605	0.016*	0.039*

Values in the table are p values (three-way ANOVA). *: Significant at 0.05 level. **: Significant at 0.01 level. ***: Significant at 0.001 level. a: ANOVA performed for Guinea Bissau, Guinea and Sierra Leone. b: ANOVA performed for Guinea Bissau, Guinea, Ghana and Togo. c: ANOVA performed for Guinea Bissau, Ghana, Sierra Leone and Togo. d: ANOVA performed for Ghana, Guinea and Togo. e: ANOVA performed for Ghana and Togo. f: ANOVA performed for all five countries. -: not assessed.

**Table 10:** Main effects of and interactions between genotype, sowing date and trial location regarding crop characteristics, including maximum canopy development (V_max_), accumulated canopy (A), plant height, number of tillers per plant (# Tillers), days to 50% flowering (50% Flowering), number of panicles per plant (# Panicles), panicle length, panicle weight, 200 grain weight and yield of the cluster of *Japonica* from Sierra Leone (Jap_SL).

Variable	Genotype	Sowing	Location	Genotype × Sowing	Genotype × Location	Sowing × Location	Genotype × Sowing × Location
**V_max_^d^**	0.433	0.293	0.097	0.526	0.461	0.133	-
**A ^d^**	0.550	0.473	0.128	0.578	0.306	0.044*	-
**Plant height ^f^**	0.072	0.568	0.003**	0.736	0.005**	0.005**	0.845
**# Tillers ^f^**	0.062	0.747	0.049*	0.775	0.072	0.023*	0.949
**50% Flowering ^f^**	0.067	0.305	0.002**	0.044*	0.069	0.037*	0.052
**# Panicles ^a^**	0.199	0.812	0.218	0.880	0.125	0.088	0.816
**Panicle length ^a^**	0.032*	0.988	0.229	0.251	0.006**	0.020*	0.637
**Panicle weight ^e^**	0.977	0.634	-	0.917	0.673	0.728	0.082
**200 grain weight ^b^**	0.328	1.000	-	0.735	0.948	0.925	0.067
**Yield ^c^**	0.114	0.082	0.619	0.516	0.943	0.422	0.000***

Values in the table are p values (three-way ANOVA). *: Significant at 0.05 level. **: Significant at 0.01 level. ***: Significant at 0.001 level. a: ANOVA performed for Guinea Bissau, Guinea and Sierra Leone. b: ANOVA performed for Guinea Bissau, Guinea, Ghana and Togo. c: ANOVA performed for Guinea Bissau, Ghana, Sierra Leone and Togo. d: ANOVA performed for Ghana, Guinea and Togo. e: ANOVA performed for Ghana and Togo. f: ANOVA performed for all five countries. -: not assessed.

Table 11 shows the average performance of the studied genotypes (grouped into botanical groups and molecular clusters) for the ten variables used. Table 12 shows the yield and yield components averaged for the five countries, whereas Table 13 shows the estimates of the wide sense heritability for the ten variables listed in Tables 1–11.

**Table 11:** Average performance of several clusters of rice (including three botanical groups and six related molecular clusters) for main crop characteristics, including maximum canopy development (V_max_), accumulated canopy (A), plant height, number of tillers per plant (# Tillers), days to 50% flowering (50% Flowering), number of panicles per plant (# Panicles), panicle length, panicle weight, 200 grain weight and yield in five West African countries.

Botanical groups and Clusters*	V_max_ (%)	A (%d)	Plant height (cm)	# Tillers	50% Flowering (d)	# Panicles	Panicle length (cm)	Panicle weight (g)	200 grain weight (g)	Yield (kg/ha)
*Glaberrima*	46.1 C	2908 B	101.1 B	6.8 B	97.1 A	6.4 C	23.4 B	2.0 A	4.3 A	1349 C
*Indica*	41.7 B	2889 B	97.8 A	7.6 C	108.9 C	5.5 B	22.1 A	1.9 A	4.1 A	757 A
*Japonica*	35.0 A	2269 A	97.2 A	4.0 A	101.8 B	2.8 A	22.5 A	3.1 B	4.3 A	967 B
Glab_UpperCoast	44.5 bcd	2794 bcd	104.2 de	6.5 c	96.7 b	6.2 cd	23.9 b	2.1 b	4.1 ab	1376 cd
Glab_LowerCoast	50.2 d	3214 d	92.7ab	7.5 d	98.4 bc	7.2 d	21.9 a	1.8 ab	4.9 c	1265 bcd
Jap_GbGh	36.8 ab	2320 ab	97.0 abc	4.4 b	101.9 c	3.1 a	22.7 ab	2.9 c	4.6 bc	1095 bc
Jap_SL	31.1 a	2085 a	98.7 cd	3.3 a	107.8 d	2.2 a	22.0 a	2.9 c	3.9 a	691 a
Ind_Gc	44.2 bcd	2984 cd	104.2 de	7.7 d	110.0 d	6.2 cd	21.6 a	1.7 ab	4.5 bc	1064 b
Ind_Gh	40.0 bc	2826 cd	91.8 a	7.4 d	110.7 d	4.8 b	22.4 a	1.5 a	3.7 a	551 a

Means in a column followed by the same letter are not significantly different from each other at 0.05% (based on Tukey tests for the botanical groups and clusters separately).

* See materials and methods section for coding of the clusters.

**Table 12:** Yield and yield components for different botanical groups and countries: Average yield (kg/ha) in descending order from left to right, number of panicles per plant, number of tillers per plant and ratio between the number of panicles and the number of tillers across countries. The values for Guinea are put in the uttermost right column as the yield was not assessed.

Botanical groups and clusters*		Ghana	Sierra Leone	Togo	Guinea Bissau	Guinea
*Glaberrima*	Yield	1660	1510	1164	1034	-
	Panicles	-	5.0	-	5.9	8.0
	Tillers	6.6	5.0	7.9	6.9	7.2
	Ratio		1.00		0.86	1.11
		Sierra Leone	Ghana	Togo	Guinea Bissau	Guinea
*Indica*	Yield	1248	1132	329	317	-
	Panicles	4.5	-	-	4.9	7.2
	Tillers	4.7	6.3	9.3	8.2	8.3
	Ratio	0.96			0.60	0.88
		Ghana	Sierra Leone	Guinea Bissau	Togo	Guinea
*Japonica*	Yield	1513	1061	759	504	-
	Panicles	-	2.9	2.6	-	3.0
	Tillers	4.9	2.9	5.1	4.0	3.5
	Ratio		0.98	0.52		0.86
		Ghana	Sierra Leone	Togo	Guinea Bissau	Guinea
Glab_UpperCoast	Yield	1664	1568	1160	1100	-
	Panicles	-	5.1	-	5.5	7.8
	Tillers	6.5	5.1	7.5	6.4	6.9
	Ratio		1.01		0.86	1.13
		Ghana	Sierra Leone	Togo	Guinea Bissau	Guinea
Glab_LowerCoast	Yield	1651	1356	1174	872	-
	Panicles	-	4.7	-	7.0	8.6
	Tillers	6.7	4.7	9.0	8.1	8.2
	Ratio		1.00		0.87	1.06
		Ghana	Sierra Leone	Guinea Bissau	Togo	Guinea
Ind_Gc	Yield	1699	1476	553	529	-
	Panicles	-	4.4	5.4	-	8.8
	Tillers	6.4	4.6	7.8	9.4	8.7
	Ratio		0.96	0.69		1.02
		Sierra Leone	Ghana	Togo	Guinea Bissau	Guinea
Ind_Gh	Yield	1096	742	196	153	-
	Panicles	4.6	-	-	4.5	5.7
	Tillers	4.9	6.3	9.2	8.5	7.9
	Ratio	0.95			0.53	0.72
		Ghana	Sierra Leone	Guinea Bissau	Togo	Guinea
Jap_GbGh	Yield	1741	1123	869	662	-
	Panicles	-	2.9	2.9	-	3.6
	Tillers	5.1	3.0	5.5	4.4	4.1
	Ratio		0.98	0.52		0.88
		Ghana	Sierra Leone	Guinea Bissau	Togo	Guinea
Jap_SL	Yield	1127	958	525	242	-
	Panicles	-	2.7	2.1	-	2.0
	Tillers	4.4	2.8	4.0	3.3	2.4
	Ratio		0.98	0.51		0.81

-: not measured. *See materials and methods section for coding of the clusters.

**Table 13:** Wide sense heritability estimates (for all genotypes together and per botanical group).

	V_max_	A	Plant height	# Tillers	50% Flowering	# Panicles	Panicle length	Panicle weight	200 grain weight	Yield per ha
All genotypes	60	45	60	79	86	77	67	75	49	76
*Glaberrima*	35	12	68	17	86	1	61	48	65	43
*Indica*	50	55	61	0	64	5	30	56	80	90
*Japonica*	76	63	45	62	59	56	69	48	32	59

### Maximum canopy cover (V_max_) and accumulated canopy cover (A)

**Figure 1:**
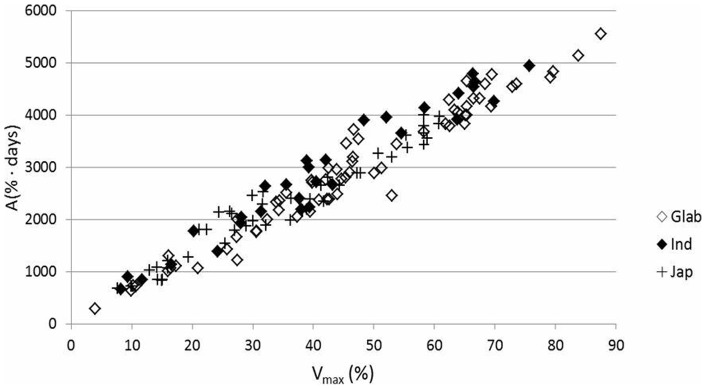
Relation between the accumulated canopy cover over the whole growing cycle (**A; y-axis, in %.days**) **and the maximum canopy cover** (**V_max_; x-axis, in %**)**.** Data refer to all combinations of location × genotype × sowing time, whereas different symbols refer to different botanical groups (*glaberrima*, *indica* and *japonica*).

V_max_ and A correlated positively (r = 0.984**) at 0.01 level. The same trend was observed for all botanical groups and molecular clusters in all environments (Tables 14–22; Figure 1). Accumulated canopy cover (A) can therefore represent V_max_ and vice versa. In all cases the surface under the canopy curves (A) can be conceived as a triangle with the cycle length (Te) as base and V_max_ as height. Variations in cycle length (Te), inflexion point (Tm1) and the time V_max_ was reached (T1) appear to confirm that A is linearly related to V_max_.

**Table 14:** Pearson correlations between yield components and days to 50% flowering.

Cluster	Plant height (cm)	Panicle length (cm)	Number of tillers	Number of panicles	Panicle weight (g)	200 grain weight (g)	Plot yield (kg/ha)	Canopy cover A (%)
All	−0.390^**^	0.073	−0.018	0.045	0.101	−0.581^**^	−0.298^**^	−0.661^**^
Glab	−0.194*	0.211*	0.111	0.304*	0.464^**^	−0.515^**^	0.080	−0.538^**^
Ind	−0.693^**^	0.115	0.413^**^	0.355	−0.306	−0.839^**^	−0.316	−0.855^**^
Jap	−0.593^**^	0.138	−0.432^**^	−0.029	−0.237	−0.716^**^	−0.511^**^	−0.880^**^
Glab_UpperCoast	−0.113	0.272*	0.043	0.385^**^	0.641^**^	−0.705^**^	0.266*	−0.482^**^
Glab_LowerCoast	−0.335	0.189	0.193	0.099	0.245	−0.714^**^	−0.428*	−0.668^**^
Ind_Gc	−0.751^**^	0.119	0.497*	0.589*	−0.416	−0.878^**^	−0.403	−0.854^**^
Ind_Gh	−0.649^**^	0.073	0.370	0.262	−0.221	−0.862^**^	−0.273	−0.873^**^
Jap_GbGh	−0.699^**^	0.058	−0.274	0.459*	−0.054	−0.685^**^	−0.559^**^	−0.896^**^
Jap_SL	−0.548^**^	0.289	−0.619^**^	−0.449	−0.611	−0.702^**^	−0.342	−0.877^**^

* : Significant at 0.05 level. **: Significant at 0.01 level.

**Table 15:** Pearson correlations between yield components and plant height (cm).

Cluster	Days to 50% flowering	Panicle length (cm)	Number of tillers	Number of panicles	Panicle weight (g)	200 grain weight (g)	Plot yield (kg/ha)	Canopy cover A (%)
All	−0.390^**^	0.225^**^	−0.206^**^	−0.168*	0.179	0.301^**^	0.346^**^	0.596^**^
Glab	−0.194*	0.337^**^	−0.384^**^	−0.530^**^	−0.067	0.051	0.168	0.671^**^
Ind	−0.693^**^	0.274	−0.495^**^	−0.113	0.580*	0.631^**^	0.392*	0.555^**^
Jap	−0.593^**^	0.034	0.093	−0.017	0.442*	0.348^**^	0.420^**^	0.621^**^
Glab_UpperCoast	−0.113	0.290^**^	−0.191	−0.408^**^	−0.098	0.438^**^	0.181	0.826^**^
Glab_LowerCoast	−0.335	0.152	−0.550^**^	−0.677^**^	−0.788^**^	0.359	0.020	0.796^**^
Ind_Gc	−0.751^**^	0.123	−0.583^**^	−0.673*	0.670	0.615*	0.228	0.784^**^
Ind_Gh	−0.649^**^	0.450*	−0.520^**^	0.143	0.674	0.682*	0.393	0.485
Jap_GbGh	−0.699^**^	−0.139	0.061	−0.134	0.229	0.359*	0.482^**^	0.635^**^
Jap_SL	−0.548^**^	0.323	0.300	0.254	0.727*	0.368	0.452*	0.640^**^

* : Significant at 0.05 level. **: Significant at 0.01 level.

**Table 16:** Pearson correlations between yield components and panicle length (cm).

Cluster	Days to 50% flowering	Plant height (cm)	Number of tillers	Number of panicles	Panicle weight (g)	200 grain weight (g)	Plot yield (kg/ha)	Canopy cover A (%)
All	0.073	0.225^**^	0.182^**^	0.120	0.102	−0.187*	−0.293^**^	−0.256^**^
Glab	0.211*	0.337^**^	0.107	0.023	0.731^**^	−0.542^**^	−0.338^**^	−0.355^**^
Ind	0.115	0.274	0.484^**^	0.124	−0.128	0.240	−0.767^**^	−0.132
Jap	0.138	0.034	0.192	−0.085	0.065	−0.159	−0.338^**^	−0.317*
Glab_UpperCoast	0.272*	0.290^**^	0.220	0.130	0.728^**^	−0.427^**^	−0.332^**^	−0.335*
Glab_LowerCoast	0.189	0.152	0.338	0.099	0.525	−0.319	−0.708^**^	−0.362
Ind_Gc	0.119	0.123	0.463*	−0.145	−0.488	−0.328	−0.850^**^	−0.227
Ind_Gh	0.073	0.450*	0.600^**^	0.485*	0.868	0.511	−0.664^**^	0.040
Jap_GbGh	0.058	−0.139	0.335*	0.091	0.087	−0.136	−0.450^**^	−0.319
Jap_SL	0.289	0.323	−0.142	−0.353	0.465	−0.379	−0.313	−0.479

* : Significant at 0.05 level. **: Significant at 0.01 level.

**Table 17:** Pearson correlations between yield components and number of tillers.

Cluster	Days to 50% flowering	Plant height (cm)	Panicle length (cm)	Number of panicles	Panicle weight (g)	200 grain weight (g)	Plot yield (kg/ha)	Canopy cover A (%)
All	−0.018	−0.206^**^	0.182^**^	0.800^**^	−0.562^**^	0.147	−0.125	0.165*
Glab	0.111	−0.384^**^	0.107	0.815^**^	0.025	0.145	−0.328^**^	−0.130
Ind	0.413^**^	−0.495^**^	0.484^**^	0.677^**^	−0.361	0.089	−0.573^**^	−0.314
Jap	−0.432^**^	0.093	0.192	0.518^**^	−0.018	0.564^**^	0.239	0.604^**^
Glab_UpperCoast	0.043	−0.191	0.220	0.768^**^	0.232	−0.137	−0.272*	−0.087
Glab_LowerCoast	0.193	−0.550^**^	0.338	0.857^**^	0.296	−0.389	−0.446*	−0.512*
Ind_Gc	0.497*	−0.583^**^	0.463*	0.895^**^	−0.527	−0.488	−0.616*	−0.532
Ind_Gh	0.370	−0.520^**^	0.600^**^	0.525*	−0.110	0.211	−0.594^**^	−0.170
Jap_GbGh	−0.274	0.061	0.335*	0.301	−0.357	0.394*	0.042	0.608^**^
Jap_SL	−0.619^**^	0.300	−0.142	0.420	0.446	0.705^**^	0.236	0.784^**^

* : Significant at 0.05 level. **: Significant at 0.01 level.

**Table 18:** Pearson correlations between yield components and number of panicles.

Cluster	Days to 50% flowering	Plant height (cm)	Panicle length (cm)	Number of tillers	Panicle weight (g)	200 grain weight (g)	Plot yield (kg/ha)	Canopy cover A (%)
All	0.045	−0.168*	0.120	0.800^**^	.^a^	0.282*	0.150	0.122
Glab	0.304*	−0.530^**^	0.023	0.815^**^	.^a^	0.083	−0.453^**^	−0.280
Ind	0.355	−0.113	0.124	0.677^**^	.^a^	0.638*	−0.201	0.137
Jap	−0.029	−0.017	−0.085	0.518^**^	.^a^	0.207	0.474^**^	−0.009
Glab_UpperCoast	0.385^**^	−0.408^**^	0.130	0.768^**^	.^a^	−0.335	−0.281	−0.228
Glab_LowerCoast	0.099	−0.677^**^	0.099	0.857^**^	.^a^	0.159	−0.824^**^	−0.521
Ind_Gc	0.589*	−0.673*	-0.145	0.895^**^	.^a^	−0.002	−0.677	0.478
Ind_Gh	0.262	0.143	0.485*	0.525*	.^a^	0.707	−0.022	0.314
Jap_GbGh	0.459*	−0.134	0.091	0.301	.^a^	−0.116	0.038	0.076
Jap_SL	−0.449	0.254	−0.353	0.420	.^a^	0.321	0.717^**^	−0.034

a : not estimated.

* : Significant at 0.05 level. **: Significant at 0.01 level.

**Table 19:** Pearson correlations between yield components and panicle weight (g).

Cluster	Days to 50% flowering	Plant height (cm)	Panicle length (cm)	Number of tillers	Number of panicles	200 grain weight (g)	Plot yield (kg/ha)	Canopy cover A (%)
All	0.101	0.179	0.102	−0.562^**^	.^a^	0.231*	0.228*	−0.225*
Glab	0.464^**^	−0.067	0.731^**^	0.025	.^a^	−0.625^**^	0.109	−0.417^**^
Ind	−0.306	0.580*	−0.128	−0.361	.^a^	0.716^**^	0.701^**^	0.503
Jap	−0.237	0.442*	0.065	−0.018	.^a^	0.379*	0.563^**^	0.251
Glab_UpperCoast	0.641^**^	−0.098	0.728^**^	0.232	.^a^	−0.553^**^	0.243	−0.268
Glab_LowerCoast	0.245	−0.788^**^	0.525	0.296	.^a^	−0.299	−0.347	−0.551
Ind_Gc	−0.416	0.670	−0.488	−0.527	.^a^	0.778*	0.755*	0.623
Ind_Gh	−0.221	0.674	0.868	−0.110	.^a^	0.617	0.702	0.574
Jap_GbGh	−0.054	0.229	0.087	−0.357	.^a^	0.563^**^	0.382	−0.046
Jap_SL	−0.611	0.727*	0.465	0.446	.^a^	0.320	0.824^**^	0.674*

a : not estimated.

* : Significant at 0.05 level. **: Significant at 0.01 level.

**Table 20:** Pearson correlations between yield components and 200 grain weight (g).

Cluster	Days to 50% flowering	Plant height (cm)	Panicle length (cm)	Number of tillers	Number of panicles	Panicle weight (g)	Plot yield (kg.ha^−1^)	Canopy cover A (%)
All	−0.581^**^	0.301^**^	−0.187*	0.147	0.282*	0.231*	0.369^**^	0.568^**^
Glab	−0.515^**^	0.051	−0.542^**^	0.145	0.083	−0.625^**^	0.218	0.596^**^
Ind	−0.839^**^	0.631^**^	0.240	0.089	0.638*	0.716^**^	0.809^**^	0.581^**^
Jap	−0.716^**^	0.348^**^	−0.159	0.564^**^	0.207	0.379*	0.621^**^	0.692^**^
Glab_UpperCoast	−0.705^**^	0.438^**^	−0.427^**^	−0.137	−0.335	−0.553^**^	0.223	0.725^**^
Glab_LowerCoast	−0.714^**^	0.359	−0.319	−0.389	0.159	−0.299	0.766^**^	0.499*
Ind_Gc	−0.878^**^	0.615*	−0.328	−0.488	−0.002	0.778*	0.902^**^	0.834^**^
Ind_Gh	−0.862^**^	0.682*	0.511	0.211	0.707	0.617	0.861*	0.612*
Jap_GbGh	−0.685^**^	0.359*	−0.136	0.394*	−0.116	0.563^**^	0.600^**^	0.708^**^
Jap_SL	−0.702^**^	0.368	−0.379	0.705^**^	0.321	0.320	0.599*	0.628*

* : Significant at 0.05 level. **: Significant at 0.01 level.

**Table 21:** Pearson correlations between yield components and plot yield (kg/ha).

Cluster	Days to 50% flowering	Plant height (cm)	Panicle length (cm)	Number of tillers	Number of panicles	Panicleweight (g)	200 grain weight (g)	Canopy cover A (%)
All	−0.298^**^	0.346^**^	−0.293^**^	−0.125	0.150	0.228*	0.369^**^	0.478^**^
Glab	0.080	0.168	−0.338^**^	−0.328^**^	−0.453^**^	0.109	0.218	0.450^**^
Ind	−0.316	0.392*	−0.767^**^	−0.573^**^	−0.201	0.701^**^	0.809^**^	0.483*
Jap	−0.511^**^	0.420^**^	−0.338^**^	0.239	0.474^**^	0.563^**^	0.621^**^	0.706^**^
Glab_UpperCoast	0.266*	0.181	−0.332^**^	−0.272*	−0.281	0.243	0.223	0.476^**^
Glab_LowerCoast	−0.428*	0.020	−0.708^**^	−0.446*	−0.824^**^	−0.347	0.766^**^	0.451
Ind_Gc	−0.403	0.228	−0.850^**^	-0.616*	−0.677	0.755*	0.902^**^	0.857^**^
Ind_Gh	−0.273	0.393	−0.664^**^	−0.594^**^	−0.022	0.702	0.861*	0.137
Jap_GbGh	−0.559^**^	0.482^**^	−0.450^**^	0.042	0.038	0.382	0.600^**^	0.848^**^
Jap_SL	−0.342	0.452*	−0.313	0.236	0.717^**^	0.824^**^	0.599*	0.497

* : Significant at 0.05 level. **: Significant at 0.01 level.

**Table 22:** Pearson correlations between yield components and canopy cover A (%).

Cluster	Days to 50% flowering	Plant height (cm)	Panicle length (cm)	Number of tillers	Number of panicles	Panicle weight (g)	200 grain weight (g)	Plot yield (kg/ha)
All	−0.661^**^	0.596^**^	−0.256^**^	0.165*	0.122	−0.225*	0.568^**^	0.478^**^
Glab	−0.538^**^	0.671^**^	−0.355^**^	−0.130	−0.280	−0.417^**^	0.596^**^	0.450^**^
Ind	−0.855^**^	0.555^**^	−0.132	−0.314	0.137	0.503	0.581^**^	0.483*
Jap	−0.880^**^	0.621^**^	−0.317*	0.604^**^	−0.009	0.251	0.692^**^	0.706^**^
Glab_UpperCoast	−0.482^**^	0.826^**^	−0.335*	−0.087	−0.228	−0.268	0.725^**^	0.476^**^
Glab_LowerCoast	−0.668^**^	0.796^**^	−0.362	−0.512*	-0.521	−0.551	0.499*	0.451
Ind_Gc	−0.854^**^	0.784^**^	−0.227	−0.532	0.478	0.623	0.834^**^	0.857^**^
Ind_Gh	−0.873^**^	0.485	0.040	−0.170	0.314	0.574	0.612*	0.137
Jap_GbGh	−0.896^**^	0.635^**^	−0.319	0.608^**^	0.076	−0.046	0.708^**^	0.848^**^
Jap_SL	−0.877^**^	0.640^**^	−0.479	0.784^**^	−0.034	0.674*	0.628*	0.497

* : Significant at 0.05 level. **: Significant at 0.01 level.

None of the botanical groups or molecular clusters showed G×E interactions for V_max_ or A (Tables 2–10). This means that within all botanical groups and molecular clusters the varieties responded comparably for V_max_ and A across environments.

However, for all three botanical groups significant sowing × location interactions were found, in particular for *glaberrima* and *japonica*. Sowing × location interactions were highly significant for the *glaberrima* botanical group and Glab_UpperCoast but not significant for the Glab_LowerCoast cluster. Glab_LowerCoast therefore maintained better V_max_ and A across environments, since its genotypes reacted in a similar way to different environments. However, the better developed canopy did not result in a yield increase as Glab_UpperCoast yielded more than Glab_LowerCoast (Table 11).

**Figure 2:**
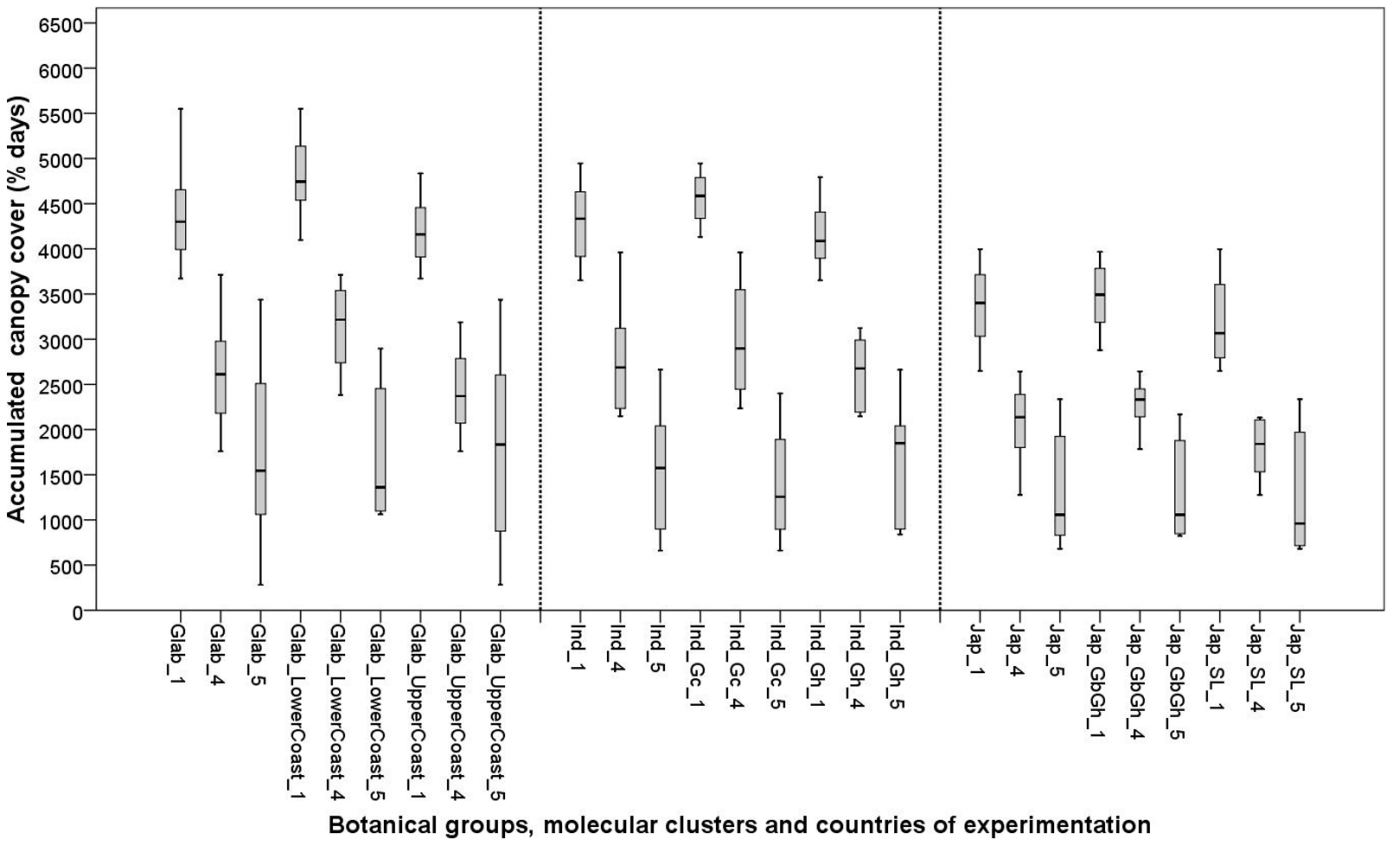
Box plots for accumulated canopy cover (**A; %.days**) **of 26 varieties in three experimental sites: Ghana** (**1**)**; Togo** (**4**) **and Guinea** (**5**)**. See materials and methods section for coding of the botanical groups and molecular clusters.**

**Figure 3:**
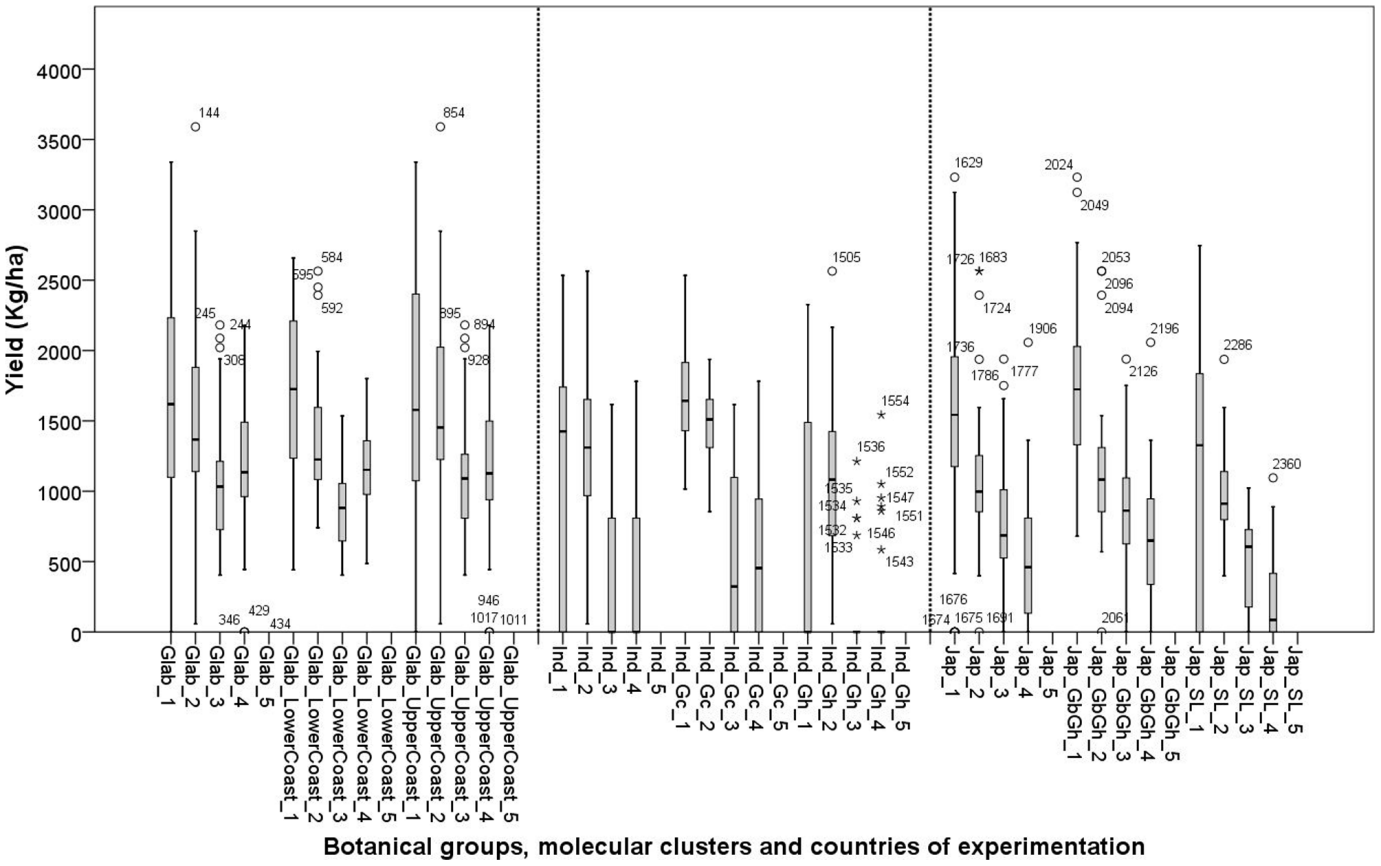
Box plots for grain yield (**in kg/ha**) **of 26 varieties in four experimental sites: 1: Ghana; 2: Sierra Leone; 3: Guinea Bissau; and 4: Togo; in 5: Guinea yield was not measured. See materials and methods section for coding of the botanical groups and molecular clusters.**

Of the *indica* group, it was only in the Ind_Gc cluster that significant sowing × location interactions were found for V_max_ and A. The *indica* group showed a significant location effect for A. No significant effects were found for the Ind_Gh cluster. This indicates that the Ind_Gh maintained better V_max_ and A than the Ind_Gc but often failed to yield (Figures 2 and 3).

The *japonica* group showed significant sowing × location interactions, suggesting that (for the two *japonica* clusters) V_max_ and A varied across environments. At cluster level significant sowing × location interactions were found for Jap_SL for V_max_ only, while for the Jap_GbGh cluster the location effects were significant for both V_max_ and A. This suggests that Jap_SL maintained A across environments better than Jap_GbGh. However Jap_SL showed considerable yield variation (Figure 3), suggesting that the relative stability observed for A did not contribute to yield stability.

Generally, the highest A was observed in Ghana followed by Togo and Guinea (Figure 2).

## Yield

The analyses of variance performed for all genotypes and at botanical group level usually showed a highly significant three-way interaction for yield (Tables 1–10). This suggests that the studied rice varieties generally responded differently in yield across environments and sowing dates. The yield variability studied at cluster level also revealed significant G×E interactions (Tables 3, 4, 6, 9 and 10) with the exception of the *indica* cluster from Guinea (Ind_Gc) (Table 7). The yield therefore varied in a similar manner across environments for genotypes of Ind_Gc.

The *glaberrima* botanical group showed the highest yields across all environments (Table 11 and Figure 3). “Zero” yields (complete crop failure) occurred only with *indica* and *japonica*. At cluster level, *glaberrima* from Upper Guinea Coast (Glab_UpperCoast) showed the highest yield. *Glaberrima* from the Lower Guinea Coast (Glab_LowerCoast) had the same yield range as *japonica* from Guinea Bissau and Ghana (Jap_GbGh) and Ind_Gc. Ind_Gh and Jap_SL showed the lowest average yield.

A comparison of the botanical groups on the yield across environments (Figure 3) shows that, within the same environment, *glaberrima* yielded more than *indica* and *japonica*. In Ghana where the average plot yield was generally high, some *indica* varieties showed “zero” yield. Zero yields occurred for *japonica* only in Guinea Bissau and Togo. These are the two countries where the overall yield was generally lowest.

**Figure 4:**
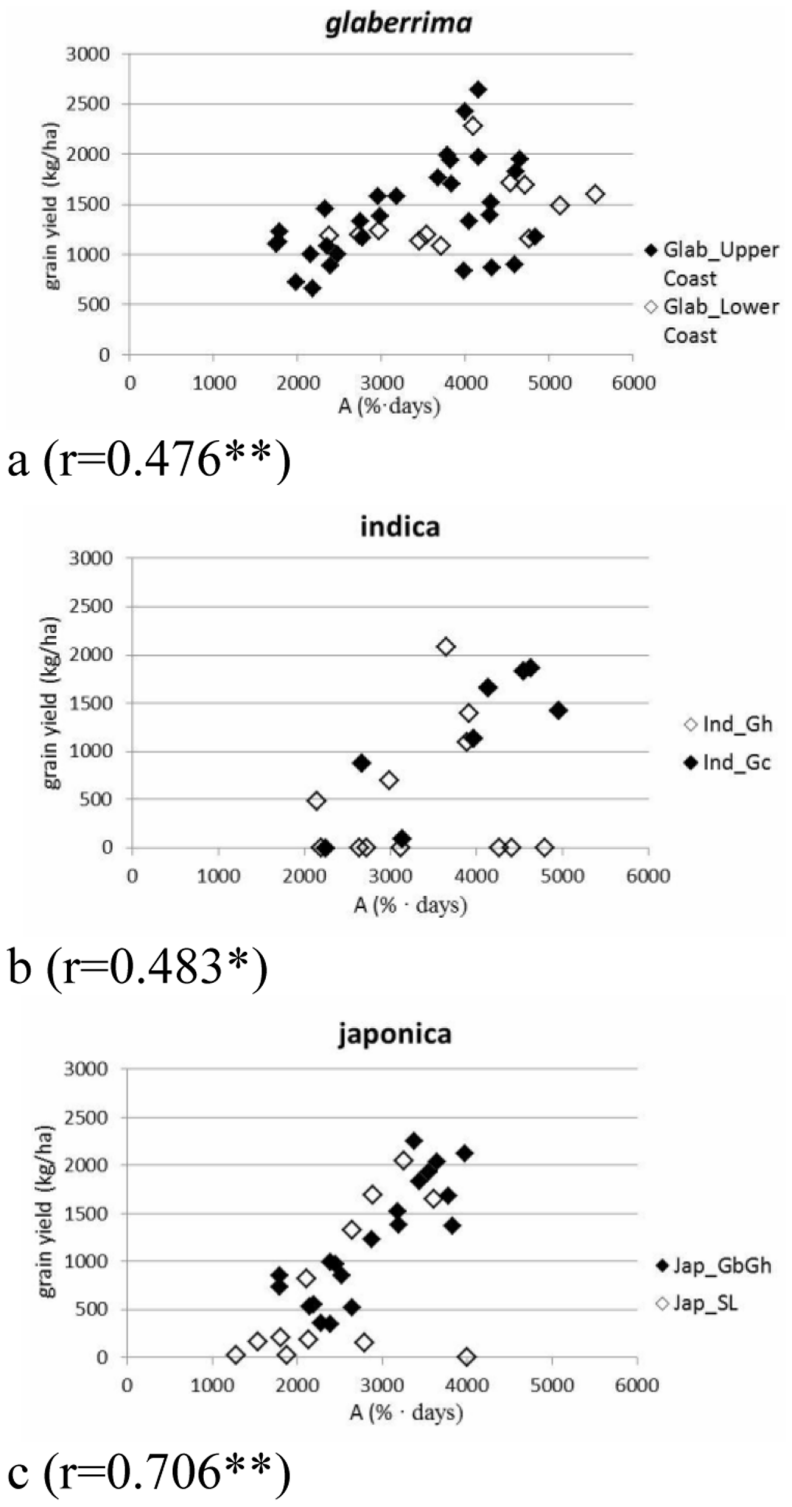
The relation between yield (**in kg/ha; y-axis**) **and accumulated canopy cover** (**A in %.days; x-axis**) **for three botanical groups.** Different symbols refer to different molecular clusters. Values presented are averages of 5 replications. Correlation coefficients are: a (varieties belonging to *glaberrima*): r = 0.476 (P<0.01); b (varieties belonging to *indica*): r = 0.483 (P<0.05); c (varieties belonging to *japonica*): r = 0.706 (P<0.01).

Figures 4a–c show the graphical representations of the relationships between yield and A for each botanical group. At cluster level different relationships were observed. The relation between yield and A was similarly low for Glab_LowerCoast and Glab_UpperCoast (r = 0.451 and r = 0.476**, respectively). This shows that *glaberrima* can yield well even when relatively low accumulated canopy cover is produced.

For the *indica* and *japonica* clusters clear differences in the relationship between grain yield and A were found. A significant relationship between yield and A was found for Ind_Gc (r = 0.857**) but not for Ind_Gh (r = 0.137). Also a significant Pearson correlation coefficient was found for Jap_GbGh (r = 0.848**) but not for Jap_ SL (r = 0.497). These findings suggest that Ind_Gc and Jap_GbGh increased their yields by producing a correspondingly dense canopy. The absence of significant correlation values for Ind_Gh and Jap_SL was caused by a number of crop failures that could be related to them being narrowly adapted to Sierra Leone only (Figures 4b and 4c).

A minimum A is indispensable for yield formation, as shown by the various associations between A and yield observed for the various clusters. But from our observation only the *glaberrima* clusters were able to yield well with low canopy development.

## Plant height

Significant G×E interactions for plant height were observed for all botanical groups and their respective clusters. This implies that across environments genotypes within botanical groups and clusters responded differently in plant height, suggesting the existence of varied strategies of adaptation for the different botanical groups and clusters. This finding confirms that plant height is in general sensitive to environmental conditions.

**Figure 5:**
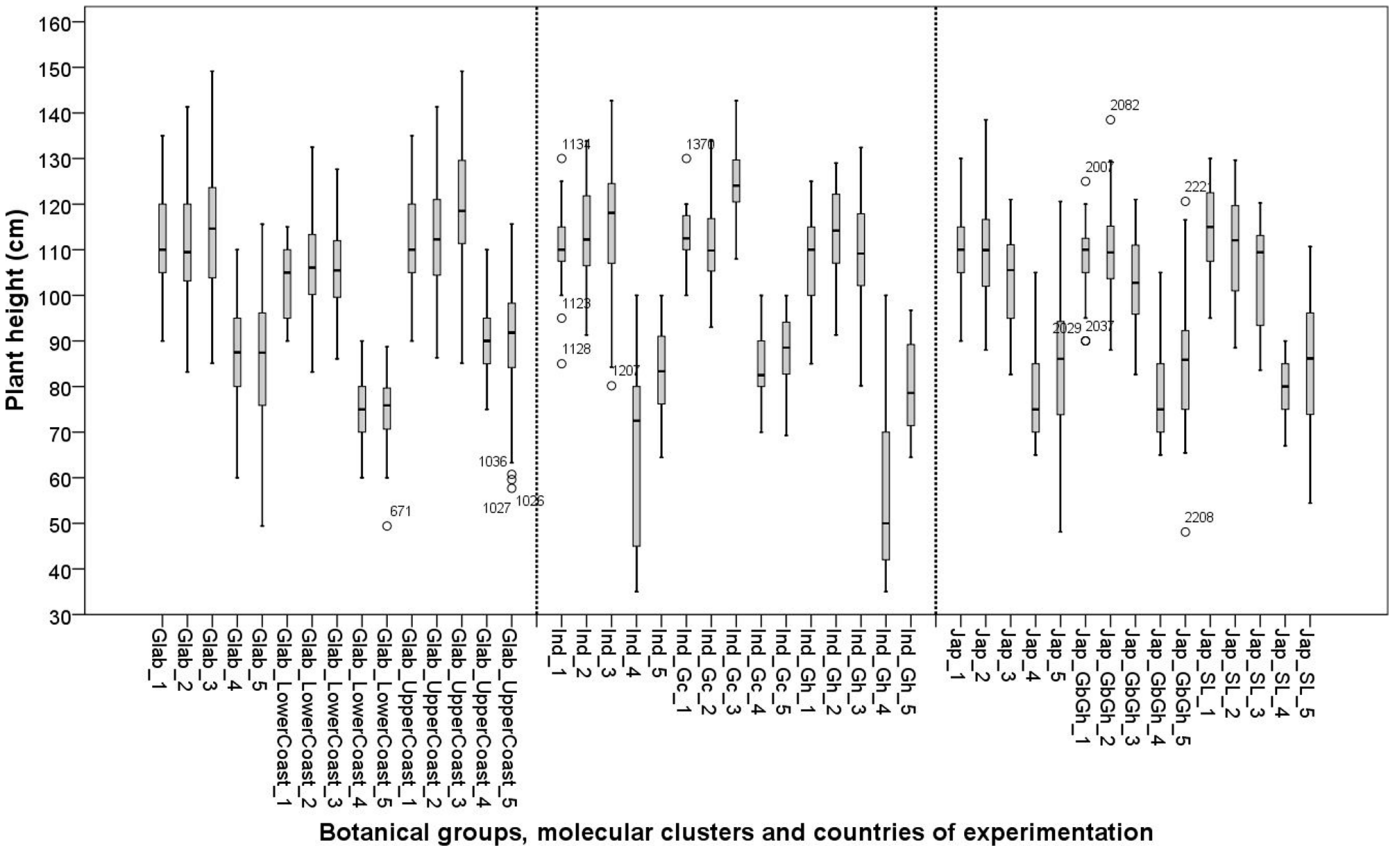
Box plots for plant height (**in cm**) **of 26 varieties in five experimental sites: 1: Ghana; 2: Sierra Leone; 3: Guinea Bissau; 4: Togo and 5: Guinea. See materials and methods section for coding of the botanical groups and molecular clusters.**

A decreasing trend was observed for plant height from countries with higher yield to countries with lower yield (Figure 5). The *O. glaberrima* group showed significantly greater average plant height than the *indica* and *japonica* groups (Table 11). At cluster level, we found that Glab_UpperCoast had taller plants than Glab_LowerCoast and that Ind_Gc had taller plants than Ind_Gh. The *japonica* clusters did not show significant differences for plant height (Table 11).

**Figure 6:**
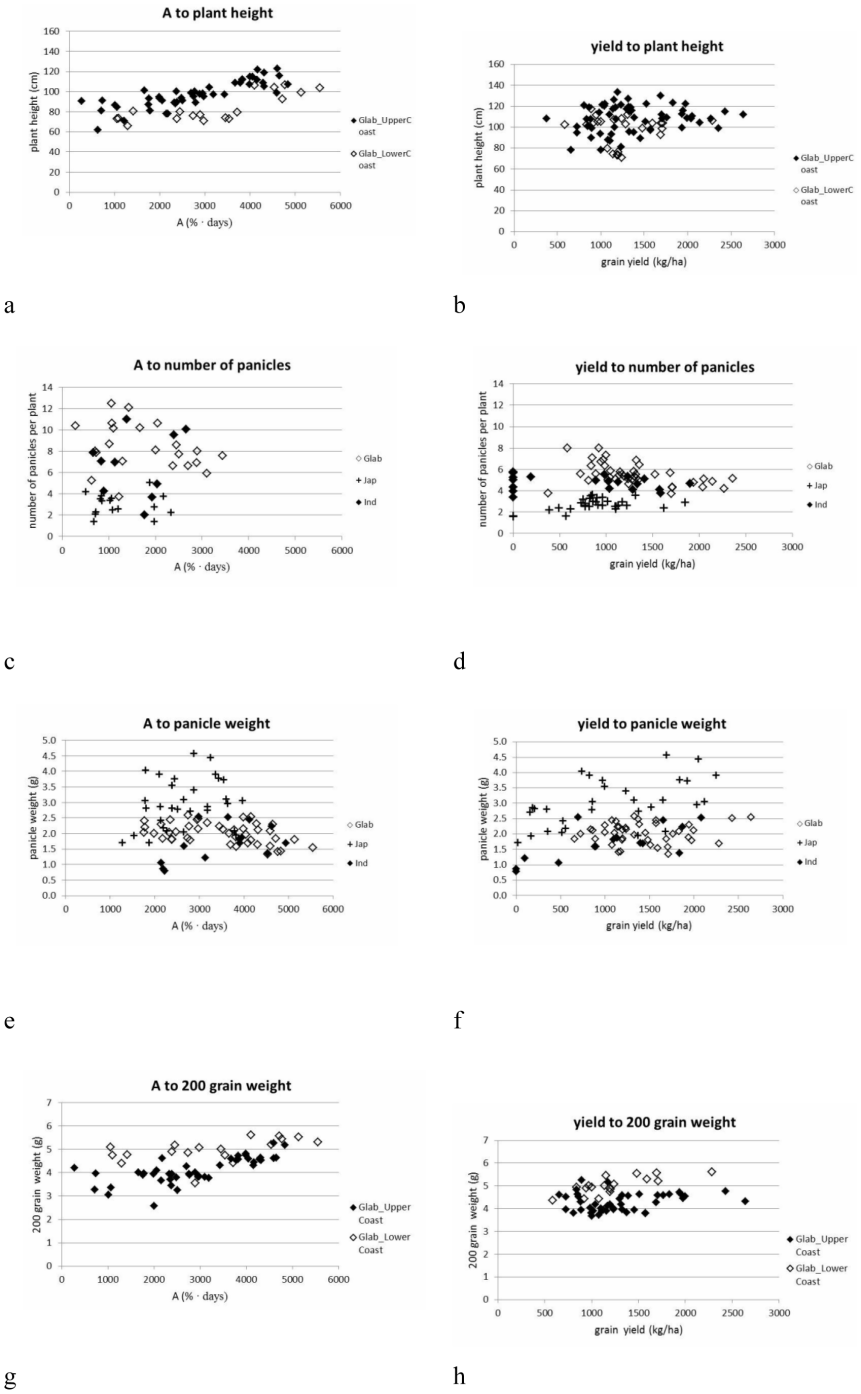
Relation between accumulated canopy cover (**A; in %.days; x-axis of a, c, e, g**) **or grain yield** (**in kg/ha; b, d, f, h**) **and plant height** (**a, b**)**, number of panicles** (**c, d**)**, panicle weight** (**e, f**) **and 200 grain weight** (**g, h**)**.** Different symbols refer to different botanical groups or molecular clusters within the *glaberrima* botanical group. Values presented are averages of 5 replications. See materials and methods section for coding of the botanical groups and molecular clusters.

**Figure 7:**
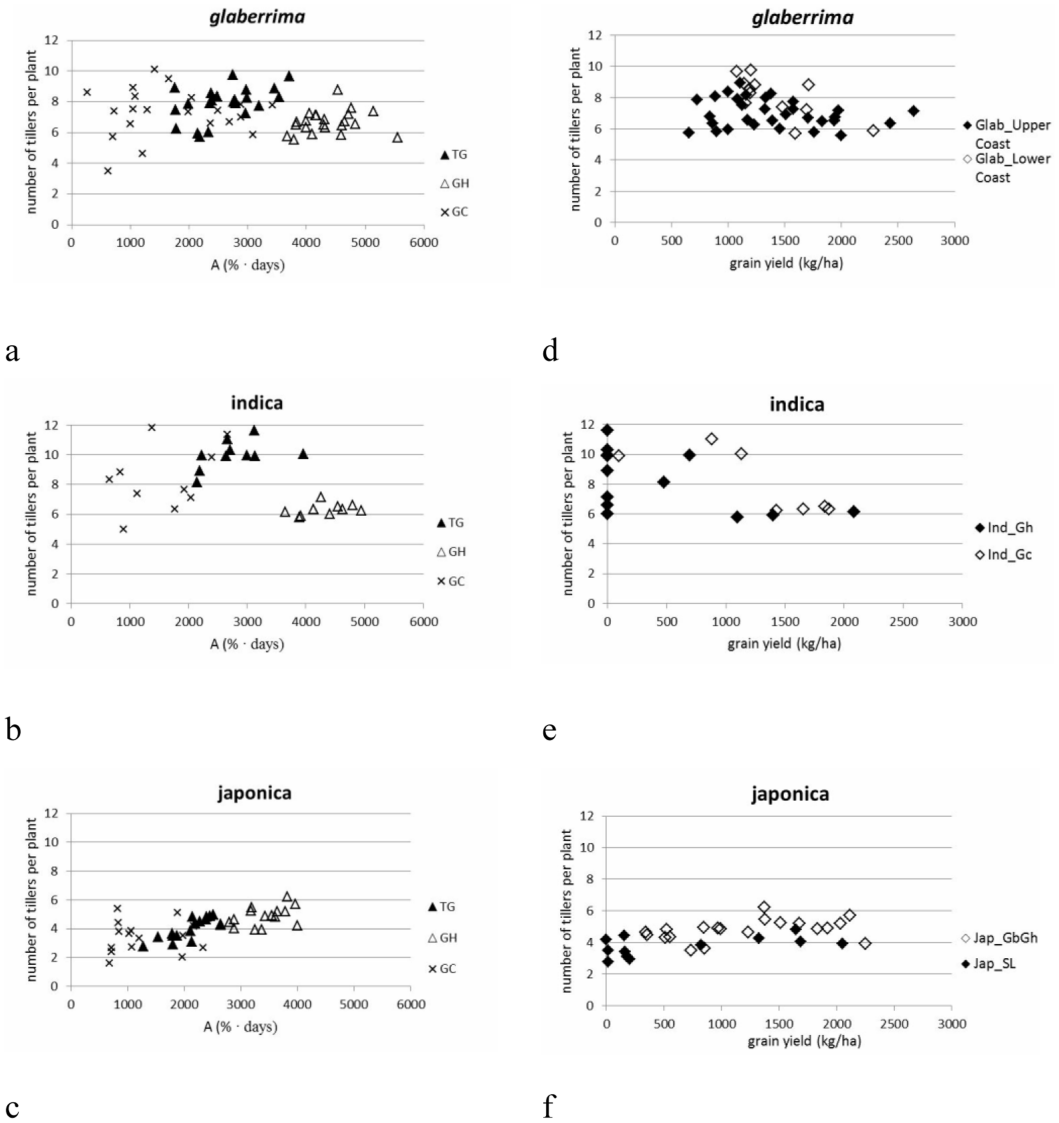
The relation between accumulated canopy cover (**A; in %.days; x-axis of a, b, c**) **or grain yield** (**in kg/ha; x-axis of d, e, f**) **and the number of tillers per plant for each of the three botanical groups and their respective molecular clusters.** Series TG, GH and GC respectively indicate observations from Togo, Ghana and Guinea. Values presented are averages of 5 replications for each of the two sowing dates. See materials and methods section for coding of the botanical and molecular clusters.

The relation between plant height and A is more strongly positive for Glab_UpperCoast (r = 0.826**, Figure 6a) than for Glab_LowerCoast. This difference is, however, absent when considering the relation between plant height and yield (Figure 6b), confirming that when more canopy was produced Glab_LowerCoast no longer invested in its height but rather in the number of its tillers, which was significantly higher for Glab_LowerCoast than for Glab_UpperCoast (Table 11, Figure 7). This suggests two distinct strategies adopted by the Glab_LowerCoast cluster and the Glab_UpperCoast cluster to arrive at similar A, and V_max_: the second cluster produces taller plants and fewer tillers and the first cluster produces shorter plants but more tillers.

Within *indica*, the cluster Ind_Gc had the tallest plants and showed a highly significant relationship between plant height and A (r = 0.784**). These observations, together with observations of high V_max_ and A for Ind_Gc, imply that Ind_Gc had a better vegetative growth compared to Ind_Gh. Cluster Ind_Gc also displayed the same average plant height as Glab_UpperCoast.

*Japonica* clusters did not show significant differences for plant height (Table 11) nor for the relationship between plant height and A: r = 0.635** and r = 0.640** for Jap_GbGh and Jap_SL, respectively.

## Number of panicles

The *glaberrima* and *indica* groups showed significant G×E interactions for number of panicles, while the *japonica* group did not (Tables 2, 5 and 8). At cluster level Glab_UpperCoast, Ind_Gh, Ind_Gc and Jap_GbGh showed significant G×E interactions (Tables 4, 6, 7 and 9). There was no such interaction for genotypes of the clusters Glab_LowerCoast and Jap_SL (Tables 3 and 10).

**Figure 8:**
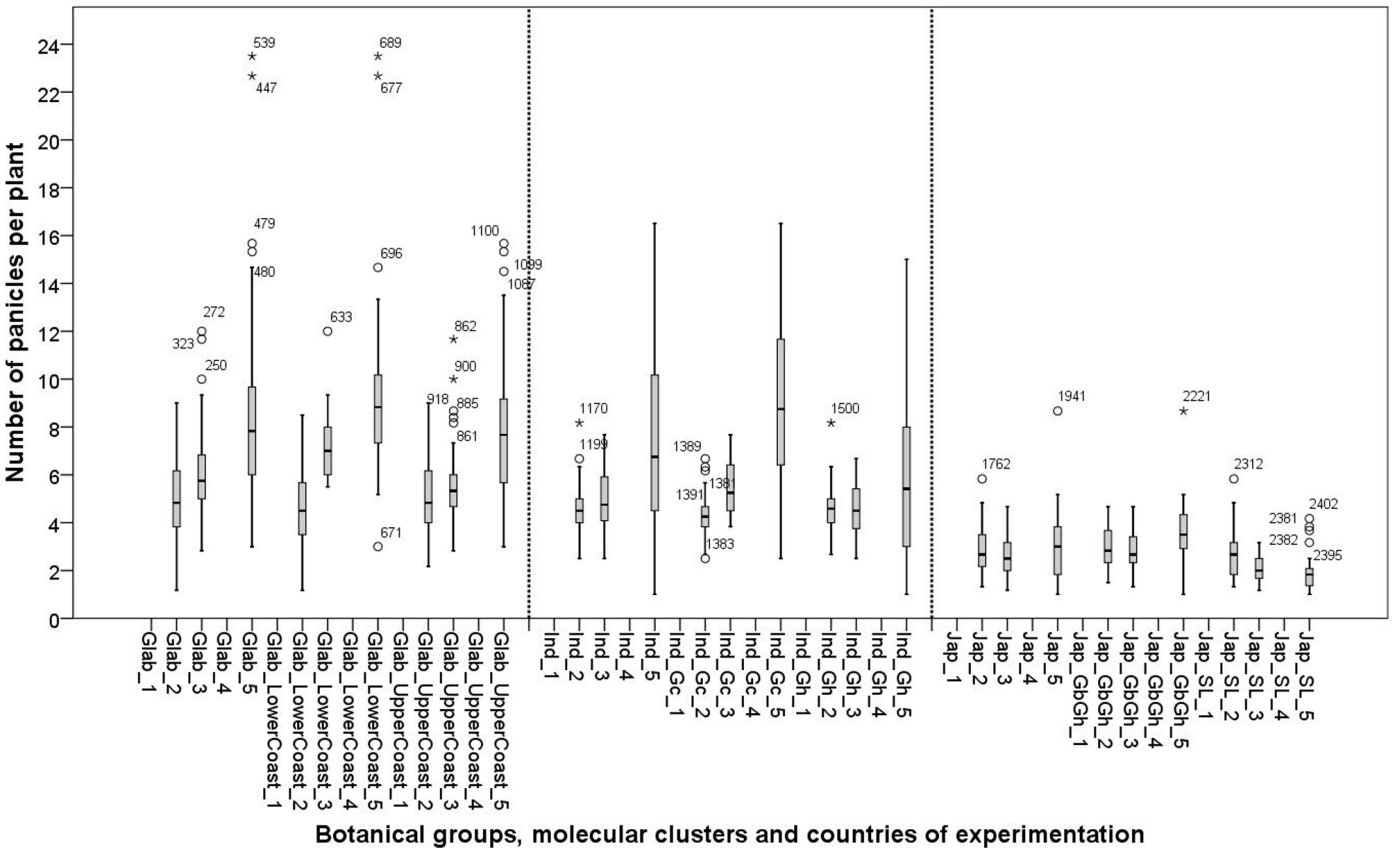
Box plots for number of panicles of 26 varieties in five experimental sites: 1: Ghana; 2: Sierra Leone; 3: Guinea Bissau; 4: Togo and 5: Guinea. See materials and methods section for coding of the botanical groups and molecular clusters.

The *glaberrima* group showed the highest average number of panicles. Cluster Ind_Gc showed a significantly higher average number of panicles than Ind_Gh and performed similar to the *glaberrima* group (Table 11). Within the *japonica* group, the highest number of panicles was observed with Jap_SL cluster in Sierra Leone, the origin of the cluster. For all botanical groups and variety clusters, the number of panicles was relatively low in Sierra Leone and Guinea Bissau and highest in Guinea (Figure 8). An opposite trend was observed only with Jap_SL. This cluster showed more panicles in Sierra Leone. This underlines our observation that Jap_SL is specifically adapted to conditions in Sierra Leone.

The *japonica* group showed the lowest numbers of panicles throughout the whole range of A and yield values (Figures 6c and 6d) and across locations (Figure 8). The number of panicles in relation to A and yield hardly overlapped for *glaberrima* and *japonica* (Figures 6c and 7d) and differed significantly (Table 11). The *glaberrima* group showed a decreasing trend in panicle number as yield values increased (r = −0.453**). For the *japonica* and *indica* groups no such decreasing trend was observed. For the *indica* group, the relation between panicle number and yield seemed to be intermediate between the tendencies for the g*laberrima* and *japonica* groups (Figure 6d), thus confirming its group distinctiveness (Table 11).

## Number of tillers

**Figure 9:**
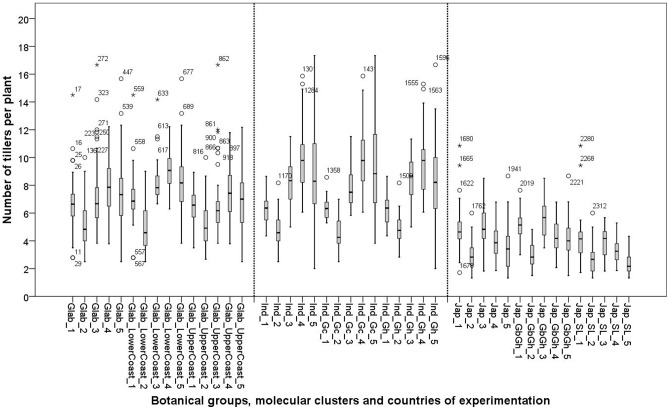
Box plots of number of tillers per plant of 26 varieties in five experimental sites: 1: Ghana; 2: Sierra Leone; 3: Guinea Bissau; 4: Togo and 5: Guinea. See materials and methods section for coding of the botanical and molecular clusters.

The three botanical groups showed significant G×E interactions for the number of tillers produced per plant. This means that, in general, genotypes composing the three botanical groups followed different strategies in tiller production across environments (Figure 9). At cluster level, G×E interactions were also found for the two *glaberrima* clusters and for the Ind_Gc cluster, but were absent for the Ind_Gh cluster and the two clusters of *japonica*. This implies that within the *japonica* clusters and the Ind_Gh cluster genotypes all vary in tiller production in a similar way across environments.

**Figure 10:**
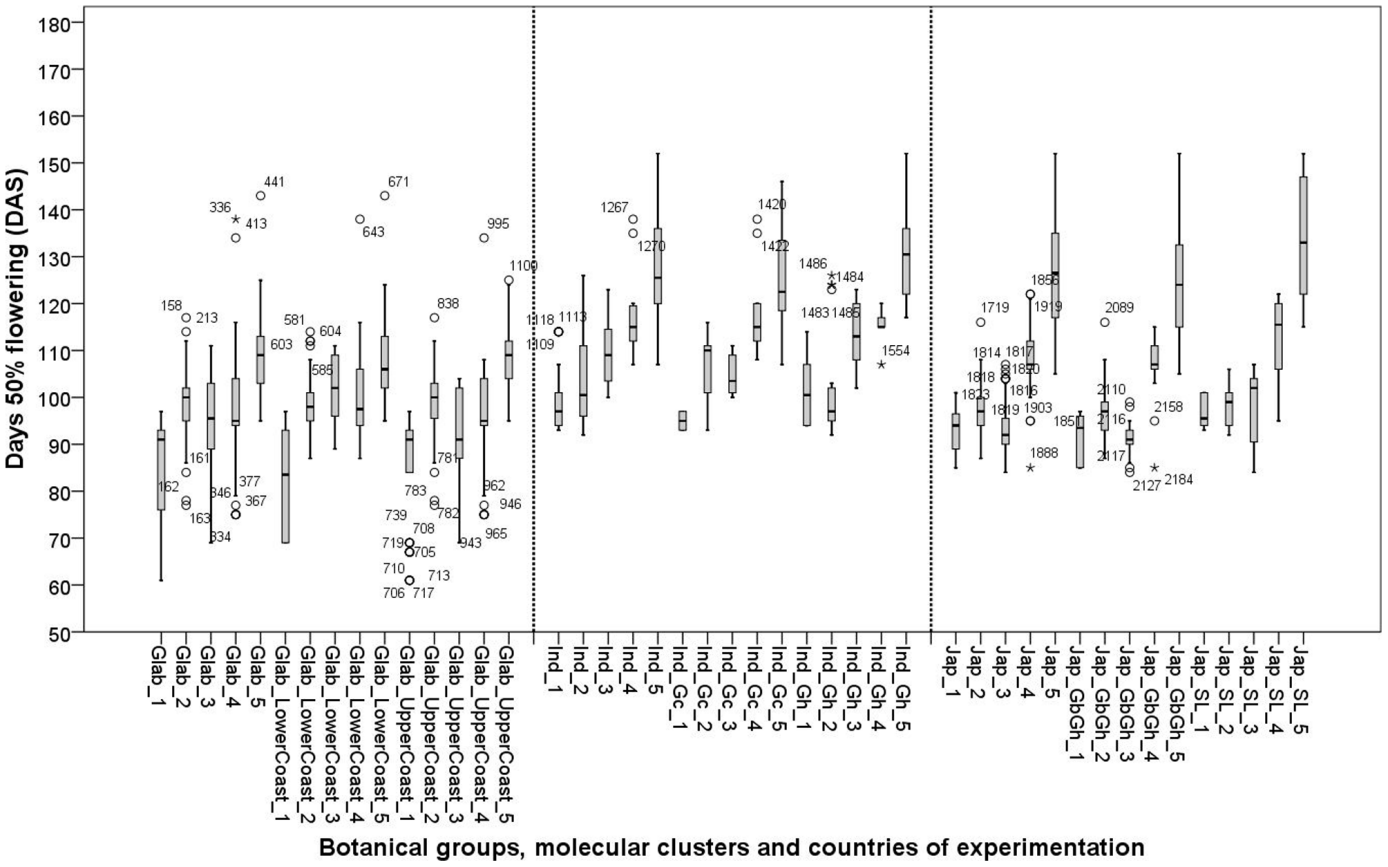
Box plots for days to 50% flowering of 26 varieties in five experimental sites: 1: Ghana; 2: Sierra Leone; 3: Guinea Bissau; 4: Togo and 5: Guinea. See materials and methods section for coding of the botanical groups and molecular clusters.

*Indica* as well as *glaberrima* showed intensive tillering (Table 11). An increase in tiller number was observed from more favourable (Sierra Leone and Ghana) to less favourable environments (Guinea, Togo and Guinea Bissau) for the *indica* cluster (Figure 9). One of the underlying mechanisms facilitating the increase of tillers under less favourable conditions is that generally (for all botanical groups and clusters) under less favourable conditions (Guinea and Togo) the time to flowering is longer than under more favourable conditions (Sierra Leone and Ghana) (Figure 10). It seems particularly the case that the *indica* group uses this time to produce tillers while the *japonica* and *glaberrima* groups responded in various other ways.

Figures 7b and 7d indicate that for the *indica* group there is a positive relationship between canopy cover and tillering in Guinea and Togo, while tillering remains constant at high A in Ghana (Figures 7b). However the positive relation in Guinea and Togo does not match with the relation between number of tillers and yield at low A because tillering remained high even when the crop failed to yield (Figure 7e).

*Japonica* showed a positive relationship between number of tillers and A (r = +0.604**, Figure 7c), but not for number of tillers and yield (Figure 7f). The two *japonica* clusters showed a similar positive relation between A and number of tillers. The Jap_GbGh cluster clearly produced more tillers than the Jap_SL cluster (Table 11). This higher number of tillers contributed to a higher panicle number (although not significantly higher) which in turn might be linked to the significantly higher yield observed for Jap_GbGh.

## Time to 50% flowering

We observed that at low yield levels the time to 50% flowering was consistently longer for all genotypes than at higher yield levels (Figure 10). This suggests that under less favourable conditions genotypes generally delayed their flowering.

## Panicle weight

Significant G×E interactions were found only for *japonica*. Sowing effects were observed for the *japonica* group (as part of the three way interaction between sowing, location and genotype), for the *indica* botanical group, and for the Ind_Gc cluster. Of the clusters only Ind_Gc showed variations in panicle weight by sowing dates. The panicle weight and yield correlated highly and positively for Ind_Gc (r = 0.755*) and Jap_SL (r = 0.824**). For other clusters no significant relations were observed between panicle weight and yield. These observations suggest that the *japonica* and *indica* groups were more sensitive to sowing date (less robust) than the *glaberrima* group and its clusters.

Panicle weight for *glaberrima* and *indica* was significantly lower than for *japonica* (Table 11). When yield and A increased, panicle weight also increased, for the *indica* group (0.549*). For the *japonica* group there was no relation between panicle weight and A. However, an increasing trend in panicle weight was observed when yield increased (0.601**) (Figures 6e and 7f). Such trends were not observed for the *glaberrima* group, suggesting that panicle weight of *glaberrima* was more stable. No significant differences or trends were found, for clusters within the *glaberrima*, *japonica* and *indica* groups, for panicle weight, with the exception of Jap_SL, which showed a positive relation with A (r = 0.674*). Panicle weight for cluster Jap_GbGh showed no relation with A.

## Panicle length

**Figure 11:**
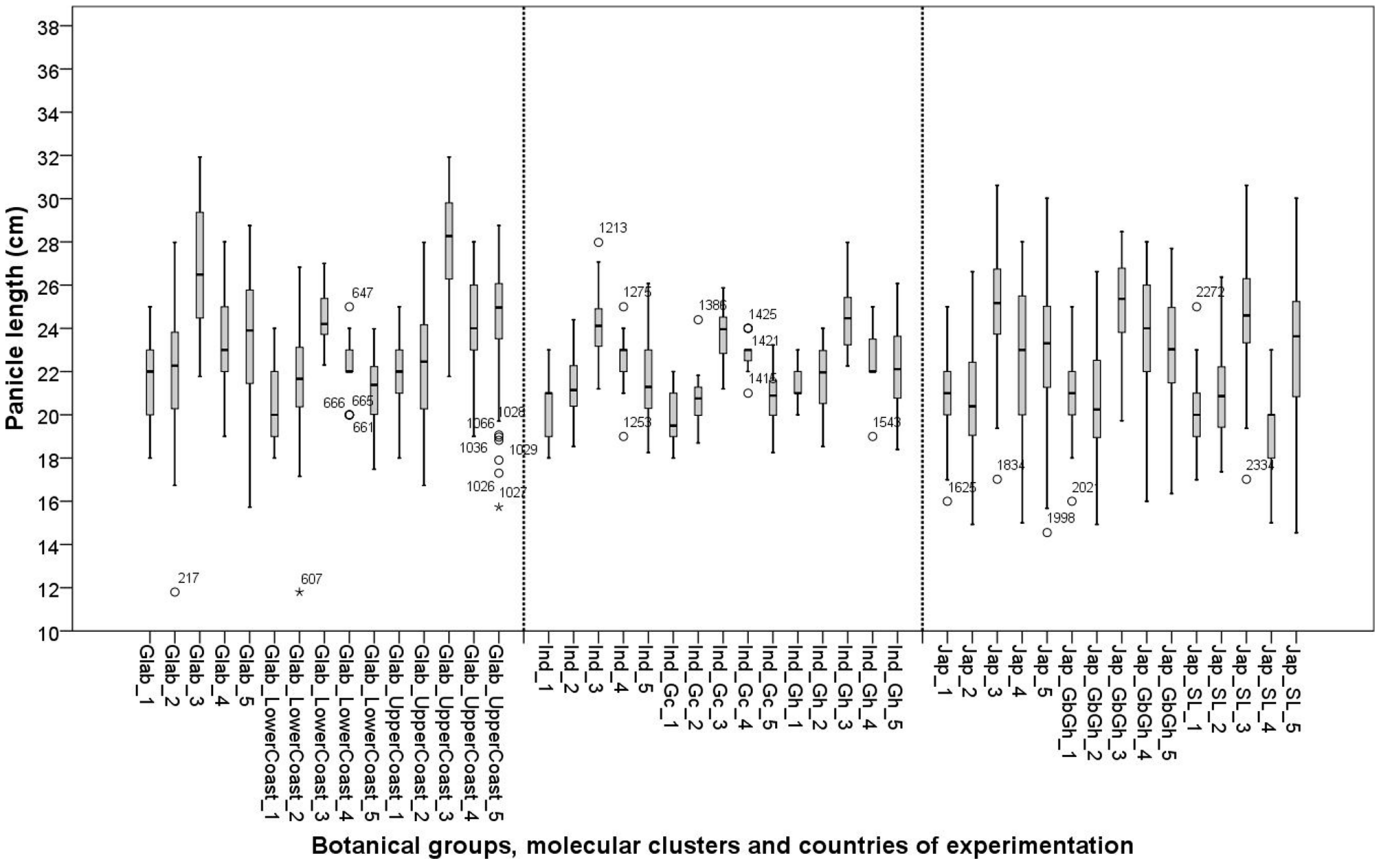
Box plots for panicle length of 26 varieties in five experimental sites: 1: Ghana; 2: Sierra Leone; 3: Guinea Bissau; 4: Togo and 5: Guinea. See materials and methods section for coding of the botanical groups and molecular clusters.

Significant G×E interactions were found for all botanical groups. The Glab_UpperCoast, Jap_GbGh and Jap_SL clusters all showed significant G×E interactions. There was a tendency towards short panicle production in Ghana and Sierra Leone, the countries where the yields were generally high (Figure 11). The cluster Glab_UpperCoast produced significantly longer panicles than all other clusters except for Jap_GbGh. The fact that the Glab_UpperCoast cluster had a panicle weight similar to that of Glab_LowerCoast implies that Glab_UpperCoast produced more grains of smaller size per panicle than Glab_LowerCoast. The cluster Glab_UpperCoast also showed a rather slight negative correlation between panicle length and yield (r = −0.332**), A (r = −0.335*) and a somewhat stronger negative correlation with the 200 grain weight (r = −0.427**). This means that for the Glab_UpperCoast, cluster production of short panicles corresponded with high A, yield and grain weight. This implies that under stress conditions (i.e. low yield and low A) Glab_UpperCoast invested more in panicle length (Figure 11). The negative relation between yield and panicle length was also observed, somewhat more strongly, for Glab_LowerCoast (r = −0.708**), Ind_Gc (r = −0.850**), Ind_Gh (r = −0.664**) and Jap_GbGh (r = −0.450**). Jap_SL did not show any relation between yield and panicle length.

## 200 grain weight

Significant G×E interactions were found for 200 grain weight for the *glaberrima* group and the Glab_UpperCoast cluster, suggesting that the genotypes composing the Glab_UpperCoast cluster responded differently across environments for 200 grain weight. This might be a factor in observed robustness in yield for this cluster. The absence of G×E interactions within the other botanical groups suggests that the 200 grain weight is genetically determined. The high estimate of wide sense heritability (H^2^ = 80%; Table 13) confirms this general trend for *indica*. However, the relatively low wide sense heritability estimate for *japonica* (H^2^ = 32%; Table 13) as compared to other botanical groups indicates that environmental conditions might have some considerable impact on the 200 grain weight of *japonica*. However, it is only with the *glaberrima* group, and not for *japonica* or *indica*, that a significant location effect was found.

Significant genotype effects were observed for the *japonica* group and the Jap_GbGh cluster. No significant genotype effect was observed for the varieties of the Jap_SL cluster, suggesting little variation for 200 grain weight in the Jap_SL cluster and large genotypic variation in the Jap-GbGh cluster. The *indica* group also showed a significant genotype effect. Not enough data were available for an ANOVA of the Ind_Gh group.

The botanical groups showed little variation for 200 grain weight, but the average 200 grain weight varied significantly among the clusters of each botanical group. Within the *glaberrima* group the Glab_UpperCoast average was lower than that of the Glab_LowerCoast cluster. The average 200 grain weight for the Jap_GbGh cluster was higher than that of the Jap_SL cluster and the Ind_Gc cluster average was higher than that of the Ind_Gh cluster.

**Figure 12:**
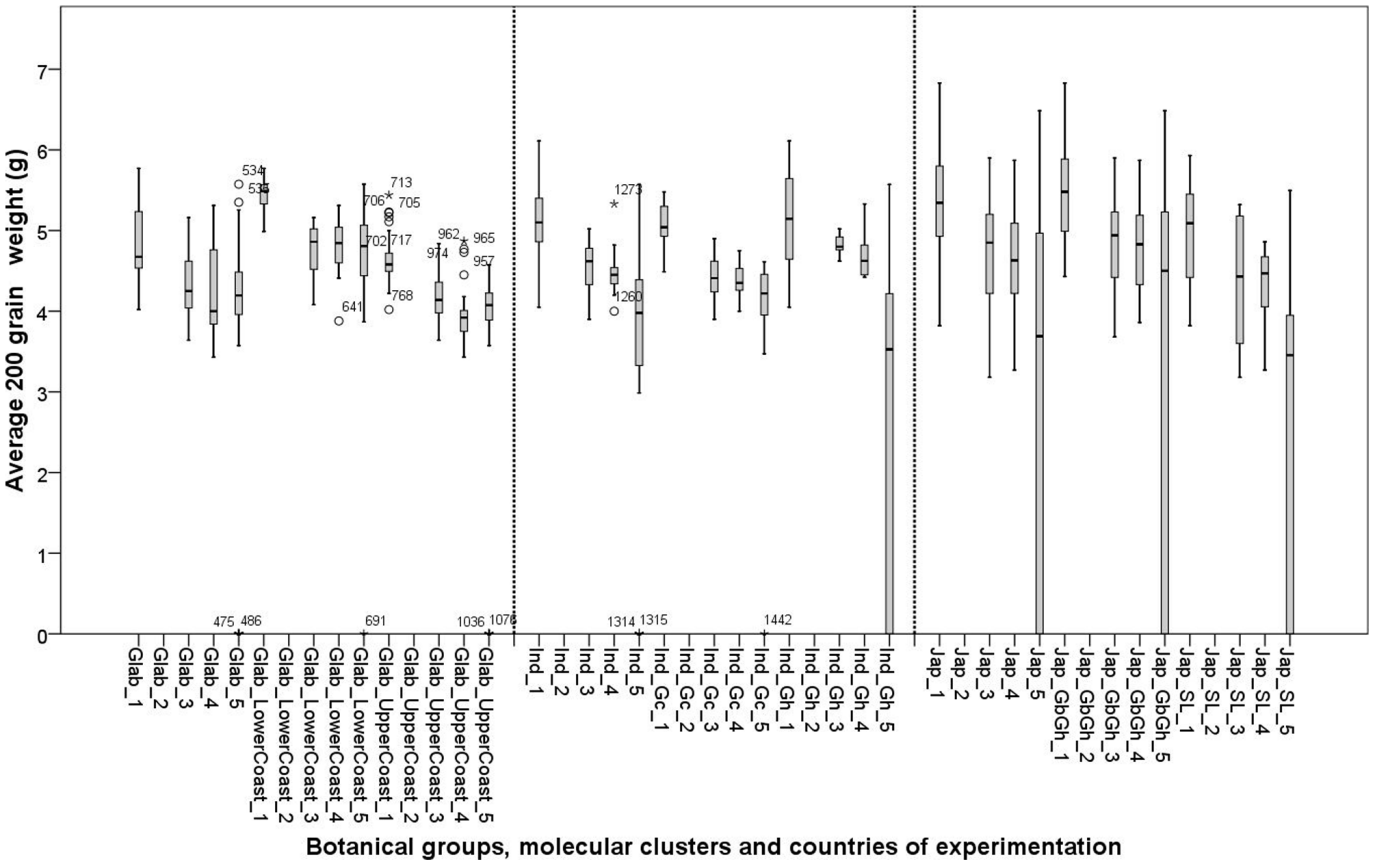
Box plots for average 200 grain weight of 26 varieties in five experimental sites: 1: Ghana; 2: Sierra Leone; 3: Guinea Bissau; 4: Togo and 5: Guinea. See materials and methods section for coding of the botanical groups and molecular clusters.

*Japonica* showed a fairly strong positive correlation between A and 200 grain weight: r = 0.70**, against r = 0.596** and r = 0.581** for the *glaberrima* and *indica* groups, respectively. At low values of A, the Ind_Gh cluster and *japonica* group tended to produce more empty or poorly developed grains, as represented in Figure 12. This is consistent with our findings under the section on number of tillers that extra tillers were produced at lower levels of A and yield contained more empty grains. The trends observed between A and 200 grain weight were also observed between 200 grain weight and yield, but only with the *indica* and *japonica* groups.

A clear divide was observed for the 200 grain values for Glab_UpperCoast and Glab_LowerCoast (Figures 6g, 6h). Figures 6g and 6 h show that when canopy cover decreased the 200 grain weight for the Glab_UpperCoast cluster decreased more than the 200 grain weight for the Glab_LowerCoast cluster. Therefore, it can be concluded that the Glab_LowerCoast cluster was less susceptible to variation in environment. The 200 grain weight for clusters within *indica* and *japonica* decreased in a similar way when A and yield decreased. These clusters were similarly sensitive to the environment. In general, all *glaberrima* clusters (and also Ind_Gc) maintained their grain weight across environments even at low yield (Figure 12). This is contrary to the Ind_Gh and two *japonica* clusters, for which the empty grains increased at lower yield levels. This underscores the claim we make for the robustness of farmer varieties of *glaberrima* and Ind_Gc, and the consequent ability of these types consistently to produce good grains throughout a range of difficult environments.

## Discussion

Figure 3 showed that the two clusters of the *glaberrima* group maintained a minimum yield of 660 kg/ha in all environments. We observed that in trials in two countries where yields were relatively high (Ghana and Sierra Leone) the *indica* sourced from Guinea maintained a yield level close to that of *glaberrima*. But in the Guinea Bissau and Togo trials, the likelihood of crop failure was high overall. This might be due to the relatively short rainy season in Guinea Bissau and to the acidity of the soil in Togo. In contrast, varieties in the Ind_Gh cluster yielded only in Sierra Leone and to a lesser extent in Ghana, with a high frequency of zero yield. In Ghana and Sierra Leone Jap_GbGh showed a yield level similar to that of the *glaberrima* clusters. In Guinea Bissau and Togo, Jap_GbGh had a low yield but still reached at least 320 kg/ha.

In contrast, Jap_SL only showed a good yield level (without zero yield) in Sierra Leone. In Guinea Bissau the yield for Jap_SL dropped to 200 kg/ha and the frequency of crop failure increased in Togo and Ghana. Jap_SL thus seemed to be specifically well adapted to the ecology of Sierra Leone. Like Jap_SL, Ind_Gh produced only in Sierra Leone. This might be attributed to the characteristics of the varieties (Viono tall and Zomojo). These varieties from Ghana are mostly cultivated in the lowlands but have proven to suit certain specific upland niches in Ghana for which the conditions were apparently not met in the Ghana trial but were approached best in Sierra Leone. Okry et al. [14] also reported on such transfer of varieties across agro-ecologies. They provided a case where farmers were trying CK 21, a typical lowland variety in the upland in the region of Guinea known as Guinea Maritime. Given that farmers have decided, for their own reasons, to shift this variety from the recommended domain, it could be counted as an instance of G×E×S (society) interaction.

These findings on the yield show that clusters differed in yield performance across environments. Glab_UpperCoast, Glab_LowerCoast, Jap_GbGh and Ind_Gc were best able to maintain their yield across environments. Farmers often look for varieties that assure minimum yield in environments with variable and stressful conditions. These varieties seemingly satisfy such objectives of farmers.

Observations of average performance at cluster level revealed that canopy development and yield scenarios differed between and within botanical groups. Glab_UpperCoast and Glab_LowerCoast showed the highest values for V_max_, A and yield. The two clusters of *indica*, Ind_Gh and Ind_Gc, showed similar values for V_max_ and A, although the latter significantly outperformed the former in yield. Moreover, Ind_Gc had a canopy development (V_max_ and A) and yield similar to Glab_LowerCoast and Jap_GbGh. Whereas Jap_GbGh and Jap_SL did not significantly differ in V_max_ or A, Jap_GbGh had a significantly higher yield than Jap_SL. Additionally, Jap_GbGh – although displaying low values of V_max_ and A – showed an average yield similar to that of *glaberrima* and Ind_Gc. The clusters Jap_SL and Ind_Gh developed a smaller canopy and also had the lowest yield. From these findings we infer that lower A can be associated with higher yield, and high canopy growth can be associated with lower yields. These associations are strongest for Ind_Gh (lower yield with higher A) and Jap_GbGh (higher yield with lower A).

Looking at the overall averages in Table 11 the ratio number of panicles over number of tillers was highest for *glaberrima* (0.94), followed by *indica* (0.72) and *japonica* (0.70), suggesting that the tillers of *glaberrima* produced more panicles. Particularly under less favourable conditions (e.g. Guinea Bissau) a difference was observed between botanical groups in the ratio of the number of panicles and tillers (Table 12). Of the botanical groups, only the clusters of the *indica* group varied, with tillers of Ind_Gc producing more panicles than those of Ind_Gh (0.80 and 0.65 respectively). However, looking at the averages per country for each botanical group and molecular cluster we observed that the increase in tillering for the *indica* group resulted in increased panicle production: the ratio of number of panicles over number of tillers remained stable or even increased at lower yield (Table 12). The combination of the high number of tillers and panicles for Ind_Gh together with low yield suggests that its panicles have a large percentage of non-formed (i.e. empty) grains.

In general the number of tillers correlated (r = 0.800**) with the number of panicles per plant which in turn correlated with A. The fact that the relationship between the number of tillers and A was not clear for all botanical groups might imply that other variables such as the size of the tillers, leaf width, leaf length and leaf blade angle, which were not measured in these experiments, might account for the overall poor relationships we observed between A and the number of tillers per plant. Vigour-related variables are known to vary between rice species, *O. glaberrima* being often more vigorous than *O. sativa* [10]–[12].

The longest average period until 50% flowering was observed with the *indica* group. The *glaberrima* group showed the shortest period until 50% flowering, suggesting that this group had a shorter vegetative cycle. The result agrees with farmers' assertions that *glaberrima* (e.g. farmer varieties Malaa and Jangjango) are often earlier than other traditional *sativa* varieties and thus are used to beat the pre-harvest hunger gap [15].

Comparing the negative relationship between time to 50% flowering and A it can be said that this relation is most clear for *japonica* and *indica* (r = −0.880** and r = −0.855** respectively). The same relation was observed at cluster level for these two botanical groups. The *glaberrima* group and its clusters showed lower correlations between 50% flowering and A (r = −0.538** for the botanical group). This might imply that the environmental conditions determining accumulated canopy cover (A) affected 50% flowering of the *glaberrima* and its clusters less than that of the other varieties. This suggests that *glaberrima* is more stable in terms of time to 50% flowering. An advantage of such stability would be that even under high stress conditions farmers do not run the risk that the crop will delay its flowering beyond the scope of the rainy season. This is more likely the case for the varieties from Upper Guinea Coast. Varieties from Lower Guinea Coast usually experience a short dry period 2 to 4 weeks after planting. In such conditions it is important for the rice crop not to flower too early. The stability in flowering time for the *glaberrim*a group takes care of that.

When summarizing the relation between the yield and yield determining variables, our study has shown that a large number of farmer varieties are able to adapt to large variations in environment. Our findings on tillering, yield, A, flowering and number of panicles suggest the existence of three different physiological strategies of adaptability for each of the botanical groups, which we now attempt to summarise.

### Glaberrima

Across environments *O. glaberrima* consistently showed the highest values for maximum canopy, plant height, number of panicles and yield. Also remarkable was the absence of crop failure for the *glaberrima* group; this helps explain why it makes a more reliable and secure choice for sub-optimal farming or situations of special difficulty. In addition, the *glaberrima* group showed the shortest time to 50% flowering, a useful property for farmers affected by a pre-harvest hunger gap [15].

Overall, accumulated canopy, maximum canopy cover and yield were similar for Glab_LowerCoast and Glab_UpperCoast clusters. But the two clusters differed in their strategy of canopy building: Glab_LowerCoast invested more in tiller production while Glab_UpperCoast produced taller plants. When A decreased, Glab_LowerCoast was better able to maintain its grain weight than Glab_UpperCoast and therefore appears to be more stable in grain weight. Under stress conditions (i.e. low yield and low A) Glab_UpperCoast invested more in panicle length. Also *glaberrima* from the Lower Coast showed higher values for 200 grain weight and the decrease of the 200 grain weight at lower yield levels was also less. However, the panicle weight for Glab_LowerCoast was less than that of the cluster Glab_UpperCoast. This also applies to panicle length and plant height. The Glab_LowerCoast varieties thus tended to invest more in grain weight, whereas Glab_UpperCoast varieties produced more grains per panicle. These two distinct strategies led to similar yields for these two clusters.

In sum, among the studied genotypes, those of *O. glaberrima* developed different strategies of adaptation, but interestingly, these strategies led to similar performance throughout the range of environments tested, demonstrating the robustness of this group of rices when compared to other botanical groups. These strategies relate to the area of collection of the varieties and also coincide with molecular groupings [16].

The *glaberrima* showed more G×E interactions than *indica* and *japonica*. This is worthy of note, since it is sometimes assumed that *O. glaberrima* is genetically less diverse than *indica* and *japonica*. Molecular analysis conducted by Nuijten et al. [16] showed that *glaberrima* and *japonica* were roughly similar in terms of genetic diversity: (H_e_ = 0.034; n = 66) and (H_e_ = 0.045; n = 87), respectively).

### Indica

In less favourable environments varieties of the *indica* group produced more tillers than in the more favourable environments. The underlying mechanism seems to be that under less favourable conditions flowering is delayed and at the same time the tillering period is prolonged. The result is that at higher yield levels *indica* produced fewer tillers. At lower yield levels *indica* seemed less vigorous, as the increase in number of tillers did not lead to an increase in A. These tillers were, however, productive because an increase in tillering led to an increase in panicle production. The fact that an increase in panicle production did not lead to an increase in yield is a product of the crop failure observed for many plots in the less favourable environments, and the many panicles with unfilled grains.

The cluster Ind_Gc showed the highest plant height. This observation together with observations of high V_max_ and A for Ind_Gc implies that Ind_Gc is more vigorous compared to Ind_Gh. This vigour resulted in higher yields for Ind_Gc. The Ind_Gc cluster also displayed the same average plant height as the Glab_UpperCoast cluster.

This shows that the Ind_Gc cluster, like *glaberrima*, is able to maintain its yield. At lower yield levels, however, it follows a different physiological strategy of adaptation than *glaberrima*, as it produced the largest number of tillers. But compared to *glaberrima*, these tillers contributed less to A and contributed also less to yield maintenance, as there were high numbers of unfilled grains.

In sum, the *indica* from Guinea resembled the *glaberrima* group in several ways. Like *glaberrima* it was able to maintain its number of tillers and also increased its number of panicles at low yield levels. Like *glaberrima*, it showed significant G×E interactions that helped to stabilise A and V_max_.

### Japonica

Low canopy cover and limited tiller and panicle production seem typical for the *japonica* group. At a high level of A, *japonica* consistently produced more tillers. This relation seemed linear, as was the relation between yield and accumulated canopy, thus suggesting that an increase in tillering contributes to canopy formation and yield. In addition, *japonica* slightly increased its panicle number while tillering, A and V_max_ were not maintained at low yield levels. Instead of investing in high tiller number *japonica* invested more in panicle weight: when compared with *glaberrima* and *indica* panicle weight was approximately 50% to 100% higher.

The Jap_GbGh cluster maintained a yield across environments similar to that of the *glaberrima* group and *indica* cluster from Guinea, although it failed to maintain A at lower yield level. In contrast, varieties in the Jap_SL cluster only yielded well in Sierra Leone. This might suggest that these *japonica* varieties were highly adapted to a specific niche. In Sierra Leone, however, varieties in the *japonica* group are often found bridging an ecological gradient from lowland to upland [15].

### Observed behaviour of the studied genotypes in relation to the area of collection

#### Glab_LowerCoast

Farmers in the Togo Hills (Togo mountain ranges) in Ghana and Togo traditionally used these varieties mainly on stony hills and slopes with poor soil because political conflict and war drove them into mountainous areas, since life on the plains was too dangerous. Reliability of yield was very important in these conditions and rice was probably once the main carbohydrate crop. The data for this cluster indeed show that they are highly reliable in relation to yield. Nowadays these varieties are cultivated on the Ghanaian slopes of the Togo Hills only for ceremonial reasons, because lowland farming has been added to the local farming repertoire since the 1960s, and other crops like cassava and maize are now more important than previously [17]. Occasionally African rice is used on the Ghanaian slopes and in the lowlands of the Togo Hills when farmers are very late with sowing rice. African rice is used because of its short cycle. Farmers in the Togo Hills (Danyi Plateau) grow only African rice, which is an important secondary crop. They said they have tried other varieties but nothing works as well in the hills as the rices of the Glab_LowerCoast cluster.

#### Glab_UpperCoast

The Upper West African Coast includes two secondary centres of domestication and diversity for *O. glaberrima* [18], so we might not expect a great deal of similarity in the behaviour of genotypes collected from this region (on a transect from Senegal to Sierra Leone). When comparing the Glab_LowerCoast to Glab_UpperCoast in our experiments the differences observed within and between clusters appear to reflect the fact that rice farmers on the Upper Coast grow rice as their main staple, and work a much broader range of environments (and thus exercise a larger range of selection pressures) than the farmers in the Togo Hills. Farmers experience quite different constraints in their farming systems. In the semi-arid zone of the Upper Coast (Senegal, Gambia and Guinea Bissau), a short rainy seasons (3 to 4 months) may have forced farmers to select for short duration *glaberrima* types better adapted to their conditions. In these conditions, farmers appear to have selected taller plants with longer panicles and fewer tillers.

In the forest belt of Sierra Leone and Guinea, with a much longer rainfall period (6 to 7 months) the environment is favourable for longer duration crops. However, farmers still cultivate *O. glaberrima* to some extent because of its adaptability to poor, eroded soils and tolerance to drought at the beginning and end of the rainy season. In the forest belt farmers report many weed problems [15], particularly in areas with short fallow periods. Selecting for tall plants could also help in suppressing weed. In addition farmers seem to have selected *glaberrima* types that were less photoperiod sensitive, facilitating the planting of short-duration types to be sown in late April and used as hunger breaker crops.

#### Ind_Gc

These varieties appeared to be stable in yield and in that way resemble *O. glaberrima* and Jap_GbGh. The Ind_Gc types are widely cultivated in the area of collection, under typical upland conditions on poor soils. Farmers state that rices in the Ind_Gc cluster resemble *O. glaberrima* in being well adapted to poor soils. They are also drought tolerant when compared to other *O. sativa* varieties (e.g. Samba, Dalifodé, Podê) and also yield well under good conditions (as well as well enough, under poor conditions). They dominate upland rice cultivation in their area of collection because, as farmers state, *O. glaberrima* lodges at complete maturity, as frequently mentioned as a drawback by a number of rice researchers [7], [19], [20]. Farmers claim this results in low yields, especially when they lack sufficient labour for a timely harvest.

#### Ind_Gh

These are varieties that performed relatively poorly in our experiments, except in Sierra Leone. In addition to cultivation under upland conditions (in the Ghanaian Togo Hills) these varieties are also cultivated very successfully in the adjacent lowlands. Since the 1960s lowland cultivation has been added to the farming systems of the different minority groups living at the foot of the Togo Hills. Ever since that time farmers have been experimenting with lowland varieties in the upland area and vice versa. The varieties in the Ind_Gh cluster are probably adapted to very specific upland conditions in the Ghanaian Togo mountain ranges, conditions apparently replicated in experimental conditions at the foot of the Sierra Leonean escarpment (Kamajei Chiefdom).

#### Jap_GbGh

These varieties are commonly planted under upland conditions. They are equal in yield to the two *O. glaberrima* clusters and the Ind_Gc cluster. Farmers grow them for their white pericarp, good taste and the fact that they fit the rainy season calendar very well, being not too short, and not too long. Farmers visiting the trial in Guinea Bissau were very impressed with the growth of some varieties of this *japonica* cluster, and indicated they would like to grow these varieties in the following season. However, upon realising the pericarp colour was red these farmers lost interest, as they have a strong preference for white seed colour. Elsewhere (in Ghana and Sierra Leone, for example) farmers actually prefer varieties with red pericarp. This underlines the importance of taking into account cultural factors in crop development [4].

#### Jap_SL

These varieties seem to be very specifically adapted to Sierra Leonean conditions. They are widely cultivated in this area of collection. Farmers who are conversant with them typically look for toposequences to allow flexible planting up and down slopes, taking account of the stage of the season. They are thus adapted to a mid-slope planting scenario, between wetland and upland varieties. The mid-slope niche is very common in an undulating, well-watered country such as Sierra Leone, but is less common in the other areas in which we carried out experiments. This may explain why this particular group only seemed to do well in its zone of collection. It has been selected for robustness in a niche.

## Conclusion

It can be concluded, that the *glaberrima* group as a whole, and the *indica* cluster from Guinea and *japonica* from Guinea Bissau and Ghana, were more plastic than other rices in the study, allowing them to be more constant in yield, A, and in number of tillers and panicles. Seemingly, farmer selection in Guinea has created a group of Asian rices that resemble in performance the highly adapted African rices of the region.

This paper has presented evidence that farmer rice varieties in coastal West Africa are, for the most part, highly robust, and well-adapted to a range of sub-optimal farming conditions. A case has been made that much of this robustness is a product of adaptation. An implication is that many farmer varieties will maintain their performance across a range of low-input conditions, and thus might be very useful to farmers in neighbouring countries. More efforts should be made to conserve, evaluate and distribute farmer-selected rice planting materials in the region. Farmers themselves should be consulted about the best way to develop relevant modalities of dissemination, and involved directly in any such activity.

## Materials and Methods

### Ethics statement

We confirm that no specific permits were required for the locations where the described field trials were conducted, that these locations were not protected in any way, and that none of these field studies involved endangered or protected species. We thank local authorities, NGOs, research institutions and farmers for their support.

### Variety collection and selection

**Figure 13:**
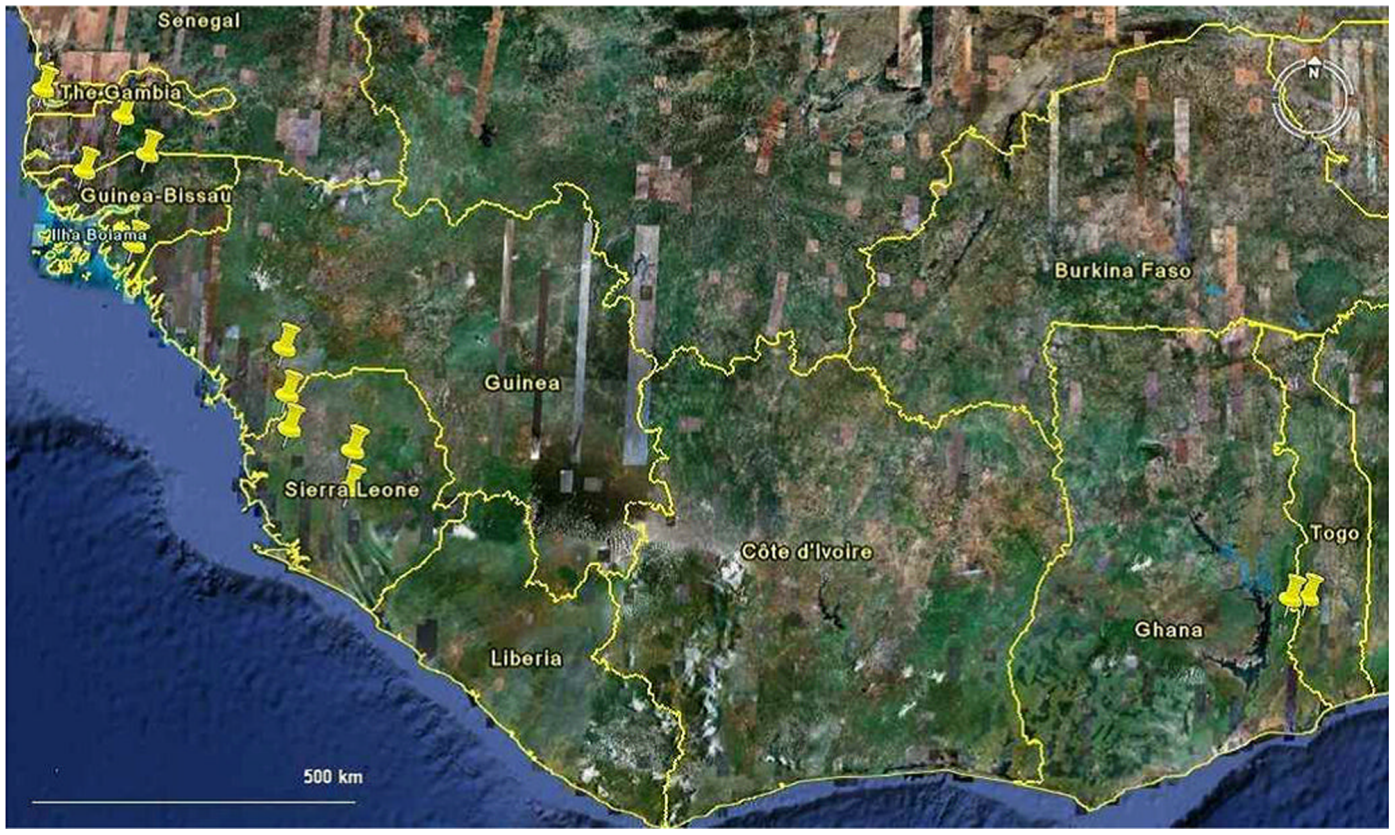
Geographic overview of the West African study area. Reprinted from [16] under a CC BY license, with permission from Edwin Nuijten, copyright 2009. Original figure generated using Google Maps.

From June to December 2007 we carried out field work in seven countries of Coastal West Africa, i.e. The Gambia, Ghana, Guinea, Guinea Bissau, Senegal, Sierra Leone and Togo (Figure 13). The field work aimed at (1) listing rice varieties/accessions used by farmers, (2) observing the development/physiology of these varieties in farmers' fields, and (3) collecting varieties at harvest. A total of 231 accessions were collected in 2007. After seed collection we carried out molecular analysis (AFLP) on the collected varieties in February and March 2008. Output of this molecular analysis was combined with the output of an analysis of 84 accessions performed in 2002 [21]. We used Version 2.2 of the software package ‘Structure’ to analyse genetic population structure and to assign samples to populations and ‘SplitsTree’ to visualize phylogenetic relationships between the samples. For further details please refer to [16]. Based on the output of the molecular analysis, 24 commonly cultivated farmer varieties (*O. glaberrima* and *O. sativa*, including representatives of both the *indica* and *japonica* groups) were selected for further study (Table 23). These 24 varieties reflect the popular varieties grown in different parts of the region and therefore provide a subset of the large set of farmer varieties identified, with good local performance but not necessarily large robustness. All 26 varieties were included in all five experiments described in this paper.

**Table 23:** List of varieties used in the study.

Code	Name of variety	Molecular cluster	Country of collection	Ecology of cultivation
*O. glaberrima*				
333	Saali Firê	Glab_UpperCoast	Guinea	Upland
347	Safaary	Glab_UpperCoast	Guinea	Upland
334	Tombo Bokary	Glab_UpperCoast	Guinea	Upland
318	Saali Forê	Glab_UpperCoast	Guinea	Upland
420	Jangjango	Glab_UpperCoast	Guinea Bissau	Upland/transition
435	Kurekimbeli	Glab_UpperCoast	Guinea Bissau	Upland/transition
113	Kaomo black	Glab_LowerCoast	Ghana (Togo mountain ranges)	Upland
124	Xleti eve	Glab_LowerCoast	Togo (Togo mountain ranges)	Upland
135	Kpakpalipke	Glab_LowerCoast	Togo (Togo mountain ranges)	Upland
272	Saliforeh	Glab_UpperCoast	Sierra Leone	Transition/upland
249	Maalay	Glab_UpperCoast	Sierra Leone	Transition/upland
*O. sativa* type *indica*				
348	Saidou Firê	Ind_Gc	Guinea	Upland
349	Saidou Gbéli	Ind_Gc	Guinea	Upland
130	Zomojo	Ind_Gh	Ghana (Togo mountain ranges)	Upland/transition/lowland
128	Viono tall	Ind_Gh	Ghana (Togo mountain ranges)	Upland/transition/lowland
163	Ataa	Ind_Gh	Ghana (Togo mountain ranges)	Upland/transition
*O. sativa* type *japonica*				
407	Demba Ba	Jap_GbGh	Guinea Bissau	Upland
427	Uyeey	Jap_GbGh	Guinea Bissau	Upland
432	Usefa Udjenel	Jap_GbGh	Guinea Bissau	Upland
141	Aqua blue	Jap_GbGh	Ghana (Togo mountain ranges)	Upland/transition
274	Nduliwa	Jap_SL	Sierra Leone	Transition/upland
210	Gbengbeng	Jap_SL	Sierra Leone	Transition/upland
215	Jebbeh-komi	Jap_SL	Sierra Leone	Transition/upland
408	Buba Njie	Jap_GbGh	Guinea Bissau	Upland/transition

Transition: variety cultivated in transitional zone between lowland and upland. Ind_Gc =  cluster of *indica* from Guinea. Ind_Gh =  cluster of *indica* from Ghana. Jap_GbGh =  cluster of *japonica* from Guinea Bissau and Ghana. Jap_SL =  cluster of *japonica* from Sierra Leone. Glab_LowerCoast =  cluster of *glaberrima* from Lower Guinea Coast. Glab_UpperCoast =  cluster of *glaberrima* from Upper Guinea Coast.

**Figure 14:**
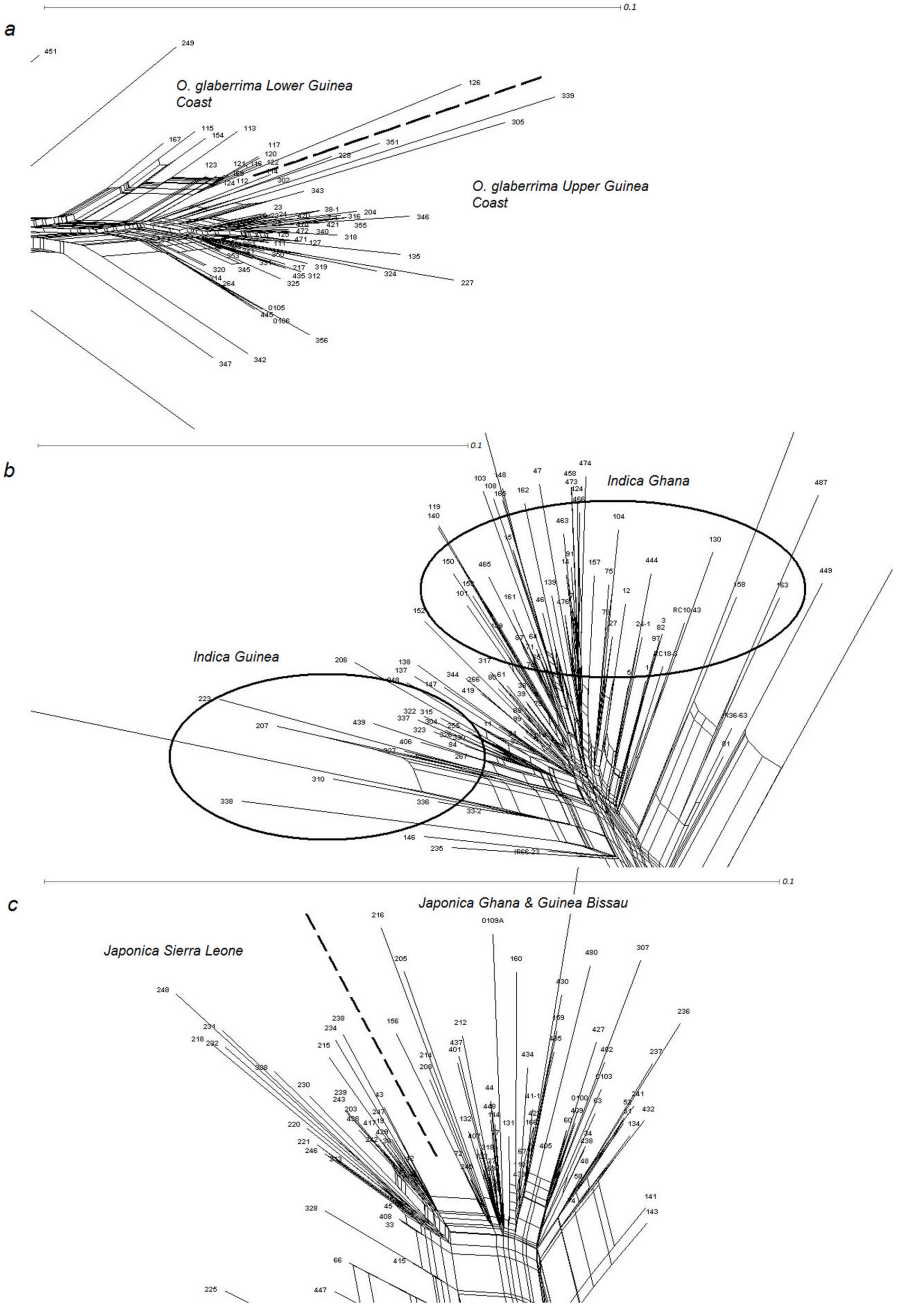
Phylogenetic relationships of *glaberrima* and its sub-clusters (**a**)**,** ***indica*** **and its sub-clusters** (**b**)**, and** ***japonica*** **and its sub-clusters** (**c**)**.**

Results of AFLP analysis suggested several clusters within the various botanical groups. These clusters were more or less coinciding with the regions where the varieties were collected. The *glaberrima* divided into a cluster from the Upper Guinea Coastal region (Glab_UpperCoast) and a cluster from the Lower Guinea Coastal region (Glab_LowerCoast) (Figure 14a). The *indica* divided into *indica* from Ghana (Ind_Gh) and *indica* from Guinea (Ind_Gc) (Figure 14b) and the *japonica* into *japonica* from Ghana and Guinea Bissau (Jap_GbGh) and *japonica* from Sierra Leone (Jap_SL) (Figure 14c). It is possible that the differences in the *japonica* group reflect different histories of introduction (Portuguese trading connections linking the Ghana and Guinea Bissau group, and British sources supplying Sierra Leone in the late 18th/early 19th centuries [22]). We used these molecular clusters in the analysis of robustness and adaptability.

### Trials

#### Locations

Five trials were conducted in Guinea, Guinea Bissau, Ghana, Togo and Sierra Leone from June 2008 to January 2009. Table 24 summarizes the characteristics of the experimental sites. Sites were selected to be representative for upland rice production on loamy soils. In all cases the experiments were planted after a fallow period.

**Table 24:** Characteristics of the experimental sites.

	Guinea	Guinea Bissau	Ghana	Togo	Sierra Leone
GPS coordinates	10.003 N, 12.918 W, 379 m asl	12.132 N, 15.936 W, 10 m asl	7.264 N, 0.470 W, 213 m asl	7.270 N, 0.716 W, 809 m asl	8.149 N, 11.908 W, 58 m asl
Ecology	Upland	Upland	Upland	Upland	Upland
Soil characteristics					
pH	4.8	4.6	4.6	4.9	4.2
OC%	2.9	1.6	1.9	5.4	4.1
Total N (g/kg)	0.9	0.2	0.7	0.9	0.6
Mehlich-3 P (ppm)	8.1	0.6	7.8	7.0	5.5
Sand (%)	69.0	81.3	63.0	65.0	16.0
Clay (%)	13.7	12.8	8.0	19.0	7.0
Silt (%)	11.1	5.3	28.0	10.0	70.0
Soil type	Sandy loam	Loamy sand	Sandy loam	Sandy (clay) loam	Silty loam
Background of experiment sites	One year fallow	At least 5 years of fallow	Five years of fallow	Three years of fallow	Twenty-four years of fallow
	Previous crops (successively): rice, groundnut (*Arachis hypogaea*), cassava (*Manihot esculenta*)		Previous crop: maize (*Zea mays*)	Previous crop: maize (*Zea mays*)	Previous crops: rice mixed cropping (cropped with squash, cucumber (*Cucumis spp.*), eggplant (*Solanum spp.*), pepper (*Capsicum spp.*), sorrel (*Hibiscus spp.*), legumes, *Zea mays*, *Manihot esculenta*, *Ipomoea batatas*, *Arachis hypogaea*, etc.
	Presence of *Imperata cylindrica*				Presence of *Pennisetum purpureum*; home for natural pests: rodents, stems borers, etc.
Average annual rainfall (mm)	2800–4000	1500	1500	1200	2100–3000
Duration rainfall (months)	6	4 to 5	7	7	6 to 7
General observations	Stress and plant mortality observed during crop establishment phase	Good germination and growth. The late maturing varieties suffered from drought and rodent damage	Most plants showed excellent germination and growth	Most plants showed some traces of acidity damage	Excellent germination and growth; low to moderate pest (rodents, termites, cut worms, stem borers) incidences were most specific to *O. sativa japonica*
Trial setup dates					
First sowing	28 June 2008	29 June 2008	16 July 2008	09 July 2008	12 June 2008
Second sowing	16 July 2008	13 July 2008	06 August 2008	30 July 2008	04 July 2008

The experiments were carried out in one growing season. By including different sowing times, we created diverse environmental conditions within each site. The growing seasons allowed normal performance of the crops, although the Guinea experiment experienced some stress during crop establishment and the Guinea Bissau experiment experienced late season drought affecting the late-maturing varieties only.

#### Experimental design

In each of the five trials, the varieties were sown in a randomized block design with two sowing dates and five replications, resulting in 26×2×5 = 260 plots. All 26 varieties were included in all experiments. Sowing dates were determined by following the farmers' practices in each region. The time between the first and the second sowing was two to three weeks. Each plot was 1.5 m ×2.1 m and contained 70 pockets, spaced 30 cm between rows and 15 cm within rows. Three to five grains were sown in each pocket and pockets were thinned to one plant within four weeks after sowing.

#### Measurements

Table 25 summarises the measured variables, the methodology of assessment and the trials in which they were recorded.

**Table 25:** Measured variables and countries of measurement.

Variables	Indication on methods of measurement	Trials where variables were measured
Canopy cover	See section: Determination of the canopy cover development	Ghana, Guinea and Togo
Plant height (cm)*	Measured from the base of the plant to the tip of the panicle of the main tiller	Ghana, Guinea, Guinea Bissau, Sierra Leone, Togo
Number of tillers*	Total number of tillers per plant	Ghana, Guinea, Guinea Bissau, Sierra Leone, Togo
Days to 50% flowering	The number of days between the sowing date and the date 50% of the plants flowered	Ghana, Guinea, Guinea Bissau, Sierra Leone, Togo
Number of panicles*	Total number of panicles per plants	Guinea, Guinea Bissau, Sierra Leone
Panicle length (cm)*	Measured from the base to the tip of the panicle of the main axis	Ghana, Guinea, Guinea Bissau, Sierra Leone, Togo
Panicle weight (g)	Weight of the grains of 14 panicles	Ghana and Togo
200 grain weight (g)	Weight of 200 filled grains. Unfilled and partially filled grains were excluded	Ghana, Guinea, Guinea Bissau, Togo
Plot yield (kg/ha)	Weight of the three inner rows	Ghana, Guinea Bissau, Sierra Leone, Togo

* Measured on 6 plants randomly selected from the inner rows.

The percentage of canopy coverage was determined during the growing cycle using frames of 60 cm ×75 cm (in Togo and Ghana) and 60 cm ×45 cm in Guinea that were put in the plot and photographed from straight above. A series of about 20 photos representing a wide range of canopy cover values was analysed with Matlab 7 and DIP image [23], to allow calculation of the percentage green in a photo. Based on this calibration the percentages of canopy coverage were estimated for all photos.

### Determination of the canopy cover development

For each plot, canopy coverage curves were made on the basis of 6 to 12 measurements. As curves for the different replications showed a large variation and a block effect was not found we decided to carry out curve fitting on the average values of the five replications.

To describe the canopy development we used a modified version of the model developed by Khan et al. [24] for potato. The model of Khan et al. distinguishes three development phases for potato: the build-up phase, the phase during which the canopy cover remains constant and the decline phase. In our case, possibly because of stress the plants experienced, the canopy never reached 100% coverage, nor did it reach a plateau level maintained for any period of time. This simplified the model because the time that the maximum canopy cover was reached (t_1_) and the time it started to decline (t_2_) coincided, resulting into a two-phase model:

Phase 1
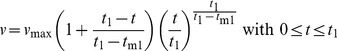
(1)


Phase 2
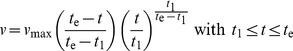
(2)where:

*v* =  canopy cover (%).

*v_max_* =  maximum canopy cover (%).

*t*_m1_ =  the inflexion point.

*t*_1_ =  the time the maximum canopy cover is reached.

*t*_e_ =  the time when the canopy has declined to 0.

*t*_m1,_ *t*_1,_ *v*_max_ and *t*_e_ were estimated using SAS.

The accumulated canopy cover A, represented by the sum of surfaces under the curves of phase 1 and 2, was estimated by using the following formulae:

Surface under the curve for phase 1 (A_1_):
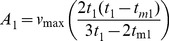
(3)


Surface under the curve for phase 2 (A_2_):
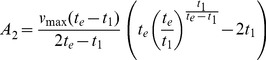
(4)


Estimation of the accumulated canopy cover (A):
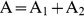
(5)


### Data analysis

### G×E interactions

As different botanical groups and molecular clusters were compared, interactions between genotypes and environment were analysed through ANOVA (analysis of variance) to assess differences in responses to different environments within and between botanical groups. Significant G×E interactions point to the presence of such a variation in response and indicate that the botanical group or cluster contains varieties that respond differently to different environments, which can be considered an indicator of adaptability within a specific botanical group or cluster. We used the Tukey test to compare means.

#### Wide sense heritability estimates

*H^2^ = 100×Vg/(Vg+1/rsVgs+1/rlVgl+1/rslVgls+1/rVe)*

where:

*H^2^*  =  wide sense heritability.

*Vg*  =  genetic variance.

*Vgs*  =  variance genetic × sowing interactions.

*Vgl*  =  variance genetic × location interactions.

*Vgls*  =  variance genetic × location × sowing interactions.

*Ve*  =  error variance.

*r* =  number of replications (5).

*s* =  number of sowings (2).

*l* =  number of locations (2, 3, 5).

#### Descriptive statistics

Averages were calculated.
